# Supplementary material for: Genome-wide association study identifies multiple new loci associated with Ewing sarcoma susceptibility
Source: Nat Commun. 2018 Aug 9;9:3184. doi: 10.1038/s41467-018-05537-2 (PMC6085378; doi:10.1038/s41467-018-05537-2)

Supplementary Information

Genome-wide association study identifies multiple new loci associated with Ewing sarcoma susceptibility

Mitchell J. Machiela, Thomas G. P. Grünewald, Didier Surdez, Stephanie Reynaud, Olivier Mirabeau, Eric Karlins, Rebeca Alba Rubio, Sakina Zaidi, Sandrine Grossetete-Lalami, Stelly Ballet, Eve Lapouble, Valérie Laurence, Jean Michon, Gaelle Pierron, Heinrich Kovar, Nathalie Gaspar, Udo Kontny, Anna González-Neira, Piero Picci, Javier Alonso, Ana Patino-Garcia, Nadège Corradini, Perrine Marec Bérard, Neal D. Freedman, Nathaniel Rothman, Casey L. Dagnall, Laurie Burdett, Kristine Jones, Michelle Manning, Kathleen Wyatt, Weiyin Zhou, Meredith Yeager, David G. Cox, Robert N. Hoover, Javed Khan, Gregory T. Armstrong, Wendy M. Leisenring, Smita Bhatia, Leslie L. Robison, Andreas E. Kulozik, Jennifer Kriebel, Thomas Meitinger, Markus Metzler, Wolfgang Hartmann, Konstantin Strauch, Thomas Kirchner, Uta Dirksen, Lindsay M. Morton, Lisa Mirabello, Margaret A. Tucker, Franck Tirode, Stephen J. Chanock and Olivier Delattre

Supplementary Tables

Supplementary Table 1. Study specific associations at each EWS susceptibility locus.

	SNP RSID	A1	A2	Beta	Std Error	Odds Ratio	95% Confidence Interval		P-value	Q	I^2
**1p36.22 (rs113663169, lead SNP)**											
Published	rs113663169	T	C	-0.80661	0.12591	0.44637	0.34875	0.57130	1.49E-10		
CCSS	rs113663169	T	C	-0.62387	0.18847	0.53587	0.37037	0.77532	0.00093		
Curie/NCI	rs113663169	T	C	-0.60890	0.18672	0.54395	0.37724	0.78432	0.00111021		
**GWAS Meta**	**rs113663169**	**T**	**C**	**-0.71642**	**0.09128**	**0.48850**	**0.40847**	**0.58420**	**4.32E-15**	**0.58**	**0.00**
**1p36.22 (rs9430161, previously published SNP)**											
Published	rs9430161	G	T	-0.78671	0.12450	0.45534	0.35675	0.58118	2.63E-10		
CCSS	rs9430161	G	T	-0.63185	0.18660	0.53161	0.36877	0.76635	0.00071		
Curie/NCI	rs9430161	G	T	-0.59957	0.18675	0.54905	0.38075	0.79173	1.33E-03		
**GWAS Meta**	**rs9430161**	**G**	**T**	**-0.70623**	**0.09055**	**0.49350**	**0.41325**	**0.58934**	**6.32E-15**	**0.64**	**0.00**
**6p25.1 (rs7742053, lead SNP)**											
Published	rs7742053	C	A	0.70623	0.13742	2.02635	1.54790	2.65268	2.76E-07		
CCSS	rs7742053	C	A	0.68448	0.19774	1.98275	1.34571	2.92135	0.000537		
Curie/NCI	rs7742053	C	A	0.22035	0.20366	1.24652	0.83625	1.85805	0.279268		
**GWAS Meta**	**rs7742053**	**C**	**A**	**0.58667**	**0.09870**	**1.79800**	**1.48175**	**2.18175**	**2.78E-09**	**0.12**	**52.80**
**6p25.1 (rs7744366, TaqMan SNP)**											
Published	rs7744366	G	A	0.62826	0.12556	1.87434	1.46546	2.39731	5.62E-07		
CCSS	rs7744366	G	A	0.69019	0.19444	1.99409	1.36217	2.91917	0.000386		
Curie/NCI	rs7744366	G	A	0.17018	0.18998	1.18552	0.81696	1.72036	0.370353		
German	rs7744366	G	A	0.40922	0.13633	1.50564	1.15259	1.96683	0.002685		
European	rs7744366	G	A	0.67937	0.11106	1.97263	1.58676	2.45233	1.00E-09		
**GWAS Meta**	**rs7744366**	**G**	**A**	**0.53427**	**0.09222**	**1.70620**	**1.42407**	**2.04422**	**6.90E-09**	**0.09**	**58.99**
**GWAS Meta+Rep**	**rs7744366**	**G**	**A**	**0.55421**	**0.06294**	**1.74056**	**1.53857**	**1.96907**	**1.30E-18**	**0.12**	**45.39**
**8q24.23 (rs7832583, lead + TaqMan SNP)**											
Published	rs7832583	T	C	0.57639	0.18324	1.77960	1.24263	2.54860	0.00166		
CCSS	rs7832583	T	C	0.76874	0.25124	2.15704	1.31826	3.52952	0.00221		
Curie/NCI	rs7832583	T	C	0.75371	0.26367	2.12486	1.26733	3.56264	0.00426		
German	rs7832583	T	C	0.06842	0.20420	1.07081	0.71763	1.59782	0.73759		
European	rs7832583	T	C	0.37411	0.13818	1.45369	1.10879	1.90588	0.00678		
**GWAS Meta**	**rs7832583**	**T**	**C**	**0.66967**	**0.12909**	**1.95360**	**1.51689**	**2.51604**	**2.13E-07**	**0.77**	**0.00**
**GWAS Meta+Rep**	**rs7832583**	**T**	**C**	**0.45041**	**0.08563**	**1.56896**	**1.32654**	**1.85569**	**1.44E-07**	**0.13**	**44.49**
**10q21.3 (rs10822056, lead SNP)**											
Published	rs10822056	C	T	0.63857	0.09451	1.89377	1.57353	2.27919	1.42E-11		
CCSS	rs10822056	C	T	0.54080	0.13837	1.71738	1.30943	2.25241	9.29E-05		
Curie/NCI	rs10822056	C	T	0.42118	0.14553	1.52376	1.14561	2.02673	0.00380		
**GWAS Meta**	**rs10822056**	**C**	**T**	**0.56588**	**0.06893**	**1.76100**	**1.53846**	**2.01573**	**1.92E-16**	**0.45**	**0.00**
**10q21.3 (rs224278, previously published SNP)**											
Published	rs224278	C	T	-0.55442	0.09357	0.57440	0.47816	0.69002	3.12E-09		
CCSS	rs224278	C	T	-0.58124	0.14136	0.55920	0.42388	0.73772	3.92E-05		
Curie/NCI	rs224278	C	T	-0.44686	0.14799	0.63963	0.47858	0.85488	2.53E-03		
**GWAS Meta**	**rs224278**	**C**	**T**	**-0.53734**	**0.06901**	**0.58430**	**0.51038**	**0.66892**	**6.88E-15**	**0.78**	**0.00**
**15q15.1 (rs2412476, lead SNP)**											
Published	rs2412476	T	C	-0.57458	0.10742	0.56294	0.45606	0.69487	8.86E-08		
CCSS	rs2412476	T	C	-0.52591	0.15598	0.59102	0.43534	0.80237	0.000747		
Curie/NCI	rs2412476	T	C	-0.50644	0.15792	0.60264	0.44221	0.82127	0.001342		
**GWAS Meta**	**rs2412476**	**T**	**C**	**-0.54645**	**0.07719**	**0.57900**	**0.49770**	**0.67358**	**1.45E-12**	**0.93**	**0.00**
**15q15.1 (rs4924410, previously published SNP)**											
Published	rs4924410	A	C	-0.46293	0.09711	0.62944	0.52034	0.76141	1.87E-06		
CCSS	rs4924410	A	C	-0.47904	0.14449	0.61938	0.46662	0.82214	9.15E-04		
Curie/NCI	rs4924410	A	C	-0.53611	0.14322	0.58502	0.44184	0.77461	1.82E-04		
**GWAS Meta**	**rs4924410**	**A**	**C**	**-0.48435**	**0.07024**	**0.61610**	**0.53686**	**0.70704**	**5.37E-12**	**0.91**	**0.00**
**20p11.22 (rs6047482, lead SNP)**											
Published	rs6047482	A	T	-0.57897	0.10976	0.56047	0.45199	0.69500	1.33E-07		
CCSS	rs6047482	A	T	-0.57231	0.16410	0.56422	0.40904	0.77828	0.00049		
Curie/NCI	rs6047482	A	T	-0.49197	0.16194	0.61142	0.44514	0.83982	0.00238		
**GWAS Meta**	**rs6047482**	**A**	**T**	**-0.55652**	**0.07950**	**0.57320**	**0.49050**	**0.66984**	**2.55E-12**	**0.90**	**0.00**
**20p11.22 (rs12106193, TaqMan SNP)**											
Published	rs12106193	G	C	-0.47853	0.12488	0.61970	0.48516	0.79155	1.27E-04		
CCSS	rs12106193	G	C	-0.73404	0.18021	0.47997	0.33714	0.68329	0.00005		
Curie/NCI	rs12106193	G	C	-0.60543	0.18299	0.54584	0.38133	0.78133	0.00094		
German	rs12106193	G	C	-0.69129	0.16708	0.50093	0.36104	0.69502	3.51E-05		
European	rs12106193	G	C	-0.43490	0.14040	0.64733	0.49160	0.85239	0.00196		
**GWAS Meta**	**rs12106193**	**G**	**C**	**-0.57199**	**0.08953**	**0.56440**	**0.47357**	**0.67265**	**1.67E-10**	**0.50**	**0.00**
**GWAS Meta+Rep**	**rs12106193**	**G**	**C**	**-0.55928**	**0.06879**	**0.57162**	**0.49952**	**0.65412**	**4.28E-16**	**0.59**	**0.00**
**20p11.23 (rs6106336, lead + TaqMan SNP)**											
Published	rs6106336	T	G	0.44871	0.13074	1.56628	1.21223	2.02374	0.00060		
CCSS	rs6106336	T	G	0.94104	0.22552	2.56265	1.64711	3.98708	3.01E-05		
Curie/NCI	rs6106336	T	G	0.49771	0.20893	1.64495	1.09221	2.47742	0.01721		
German	rs6106336	T	G	0.31438	0.14959	1.36942	1.02142	1.83597	0.03558		
European	rs6106336	T	G	0.18995	0.11849	1.20918	0.95859	1.52529	0.10892		
**GWAS Meta**	**rs6106336**	**T**	**G**	**0.55561**	**0.09947**	**1.74300**	**1.43426**	**2.11820**	**2.33E-08**	**0.16**	**45.45**
**GWAS Meta+Rep**	**rs6106336**	**T**	**G**	**0.38629**	**0.06846**	**1.47151**	**1.28674**	**1.68282**	**1.68E-08**	**0.05**	**58.10**

Supplementary Table 2. Concordance tables comparing TaqMan genotypes to array imputed genotypes. The correlation_c is the correlation between continuous imputed dosages and the TaqMan genotypes and the correlation_d is the correlation between imputed hard genotype calls (e.g. 0, 1, or 2) and TaqMan genotypes.

rs7744366

TaqMan Genotype \ Imputed Genotype	0	1	2	
0	241	7	0	248
1	1	75	0	76
2	0	0	7	7
NA	2	4	0	6
	244	86	7	337

concordance: 95.8%
correlation_c: 94.8%
correlation_d: 95.1%

rs7832583

TaqMan Genotype \ Imputed Genotype	0	1	2	
0	294	0	0	294
1	0	40	0	40
2	0	0	0	0
NA	2	0	1	3
	296	40	1	337

concordance: 99.1%
correlation_c: 100%
correlation_d: 100%

rs12106193

TaqMan Genotype \ Imputed Genotype	0	1	2	
0	213	8	0	221
1	5	90	3	98
2	0	4	12	16
NA	0	2	0	2
	218	104	15	337

concordance: 93.5%
correlation_c: 94.0%
correlation_d: 91.0%

rs6106336

TaqMan Genotype \ Imputed Genotype	0	1	2	
0	264	0	0	264
1	2	63	0	65
2	0	1	2	3
NA	3	0	2	5
	269	64	4	337

concordance: 97.6%
correlation_c: 97.8%
correlation_d: 97.6%

Supplementary Table 3. Allele frequencies of EWS risk allele across 1000 Genomes Project African (AFR), Eastern Asian (EAS) and European (EUR) populations.

Region	Top SNP	Alleles		Population Risk Allele Frequency		
		Ref	Risk	AFR	EAS	EUR
1p36.22	rs113663169	C	T	0.469	0.921	0.805
6p25.1	rs7742053	C	A	0.274	0.158	0.120
8q24.23	rs7832583	T	C	0.323	0.150	0.071
10q21.3	rs10822056	C	T	0.611	0.387	0.495
15q15.1	rs2412476	C	T	0.478	0.290	0.708
20p11.22	rs6047482	T	A	0.256	0.676	0.731
20p11.23	rs6106336	T	G	0.161	0.320	0.145

Supplementary Table 4. Distance of top SNP in EWS susceptibility regions to the nearest EWSR1-FLI1 binding site in A673 and TC71 cell lines. These variants were significantly closer to EWSR1-FLI1 binding sites than 1,000 randomly selected SNPs on chromosomes 1, 6, 10, 15, and 20 in the A673 and TC71 cell lines (Wilcoxon P-values=0.0025 and 0.0009, respectively).

**Locus**	**Top SNP**	**Distance of the closest EWSR1-FLI1 peak**	
		**A673**	**TC71**
1	1:11046565	1,031	806
6	6:6841452	3,616	3,913
10	10:64514111	24,943	56,760
15	15:40335852	1,361	3,008
20_cond	20:21055045	28,225	15,488
20	20:21539683	2,469	3,000

Supplementary Table 5. Reported dbSNP germline genetic variants in or around GGAA microsatellites in EWS susceptibility regions.

dbSNP_ID	strand	ALT (CEU)	ALT (AFR)	ALT	REF	Type	Consequence
6p25.1 Region							
rs10541084	+	0.427	0.373	GAAG	-	indel	ALT enlarges the mSat
rs747450015	+	very rare	very rare	-	GAAGGAAG	indel	REF enlarges the mSat
rs773578278	+	very rare	very rare	-	GAAG	indel	REF enlarges the mSat
rs201655383	+	very rare	very rare	A	G	SNP	REF enlarges the mSat
rs1290423	-	very rare	very rare	T	C	SNP	REF enlarges the mSat
rs183899156	+	very rare	very rare	G	A	SNP	ALT enlarges the mSat
rs1290422	-	very rare	very rare	C	T	SNP	ALT enlarges the mSat
rs186451795	+	very rare	very rare	A	G	SNP	REF enlarges the mSat
20p11.22 Region							
rs140435773	+	0.1223	0.3933	C	-	indel	REF enlarges the mSat
rs6035896	+	0.0129	0.0166	C	T	SNP	REF enlarges the mSat
rs74187972	+	very rare	very rare	C	-	indel	REF enlarges the mSat
rs536579051	+	very rare	very rare	-	CTT	indel	REF enlarges the mSat
rs57407269	+	very rare	very rare	-	TTC	indel	REF enlarges the mSat
rs199961320	+	very rare	very rare	-	TCC	indel	REF enlarges the mSat
rs955238766	+	very rare	very rare	G	C	SNP	REF enlarges the mSat
rs868358636	+	very rare	very rare	C	T	SNP	REF enlarges the mSat
rs796575942	+	very rare	very rare	T	C	SNP	REF enlarges the mSat
rs57875684	+	very rare	very rare	-	C	indel	REF enlarges the mSat
rs140662159	+	very rare	very rare	TCTT	-	indel	REF enlarges the mSat
rs4815052	+	0.9583	1.0000	T	C	SNP	REF enlarges the mSat
rs200957039	+	0.9245	0.9939	TTCT	-	indel	ALT enlarges the mSat
rs61629094	+	very rare	very rare	-	CCTTCCT	indel	REF enlarges the mSat

Supplementary Table 6. Reported eQTLs in GTEx tissues that are in linkage disequilibrium with the 20p11.22 (rs6047482) and 20p11.23 (rs6106336) GWAS lead variants. The cutoff for linkage disequilibrium was an R^2<0.5 in the CEU population. Only significant eQTLs (P-value<0.05) for the most associated GTEx tissue-specific variants in linkage disequilibrium with the lead variant are reported.

**GTEx Tissue**	**EWS GWAS Region**	**Most Associated Variant (R^2 CEU)**	**eQTL Association P-value by Gene**					
			INSM1	***LINC00261***	***NKX2-2***	***PAX1***	***KIZ****	***XRN2***
Artery - Tibial	20p11.22		n.s.	n.s.	n.s.	n.s.	n.s.	n.s.
	20p11.23	rs11087340 (1.00)	n.s.	n.s.	n.s.	n.s.	8.0E-05	n.s.
Cells - Transformed fibroblasts	20p11.22	rs112337001 (0.51)	n.s.	n.s.	n.s.	n.s.	n.s.	7.3E-06
	20p11.23		n.s.	n.s.	n.s.	n.s.	n.s.	n.s.
Skin - Sun Exposed (Lower leg)	20p11.22	rs6035892 (0.54)	n.s.	n.s.	n.s.	n.s.	4.2E-07	n.s.
	20p11.23	rs6047244 (0.72)	n.s.	n.s.	n.s.	n.s.	1.8E-08	n.s.
Testis	20p11.22	rs6035892 (0.54)	n.s.	n.s.	n.s.	n.s.	4.2E-06	n.s.
	20p11.23	rs6137252 (1.00)	n.s.	n.s.	n.s.	n.s.	5.1E-10	n.s.
Whole Blood	20p11.22		n.s.	n.s.	n.s.	n.s.	n.s.	n.s.
	20p11.23	rs6137246 (0.96)	n.s.	n.s.	n.s.	n.s.	1.4E-11	n.s.

**KIZ* is denoted as the alias *PLK1S1* in GETx
n.s.=not significant

Supplementary Table 7. Commercially available TaqMan assays used in genotype validation and independent replication.

RefSeq ID	Oligo ID	Poly ID	Sequence	Direction
**rs7744366**	Oligo-104717	Poly-0156621	TCAGGCACCAAATTCCTAAGCA	F
	Oligo-104718	Poly-0156621	ACTCTGATGGACCTGCAGGA	R
	Oligo-104719	Poly-0156621	CCCTTCCTACAGGACAG	
	Oligo-104720	Poly-0156621	CCCTTCCTACGGGACAG	
**rs7832583**	Oligo-104705	Poly-0156616	GTGGTACTAGAAATTGACAATGTGCAT	F
	Oligo-104708	Poly-0156616	CCTAGGGAACAGAGGGAGACT	R
	Oligo-104711	Poly-0156616	AATAAAGCTTAGGGATATAA	
	Oligo-104714	Poly-0156616	AGAAATAAAGCTTAGAGATATAA	
**rs12106193**	Oligo-106123	Poly-0156651	GTGCATCCCTGGTCCTTCT	F
	Oligo-106125	Poly-0156651	CACATGAGACCCAGTGAAACCAT	R
	Oligo-106127	Poly-0156651	TTGTGAAGCCTCTTTC	
	Oligo-106129	Poly-0156651	TTGTGAAGGCTCTTTC	
**rs6106336**	Oligo-104707	Poly-0156618	GGGACAGTCAGAAGAAATTCAGAAGA	F
	Oligo-104710	Poly-0156618	AGCAAGAGATGCACCAGTAATGG	R
	Oligo-104713	Poly-0156618	CCTCTGGACTTTGAC	
	Oligo-104716	Poly-0156618	CCTCTGGAATTTGAC	

Supplementary Figures

Supplementary Figure 1. Quantile-quantile plot of expected $-\log_{10}$ P-values to observed $-\log_{10}$ P-values. Dashed line is where observed equals expected. A total of 6,876,682 variants were included in the meta-analysis with a resulting lambda statistic of 1.0354.

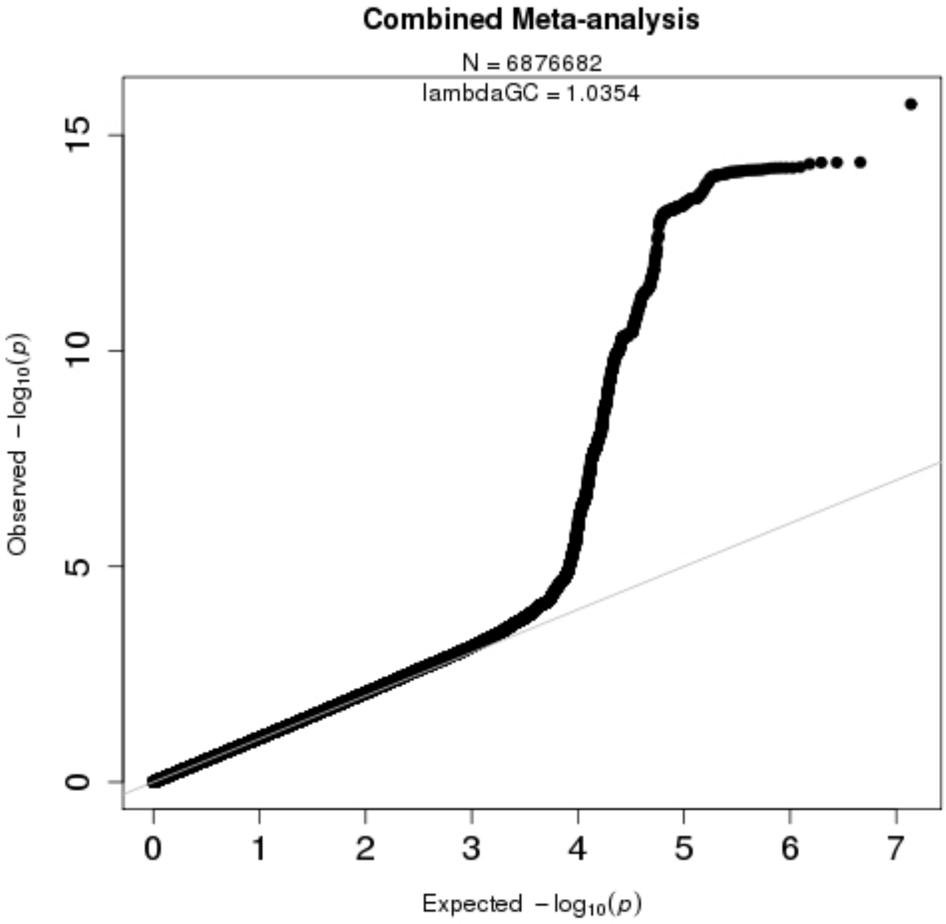

Supplementary Figure 2A. Meta-analysis forest plots for the 1p36.22 EWS susceptibility locus.

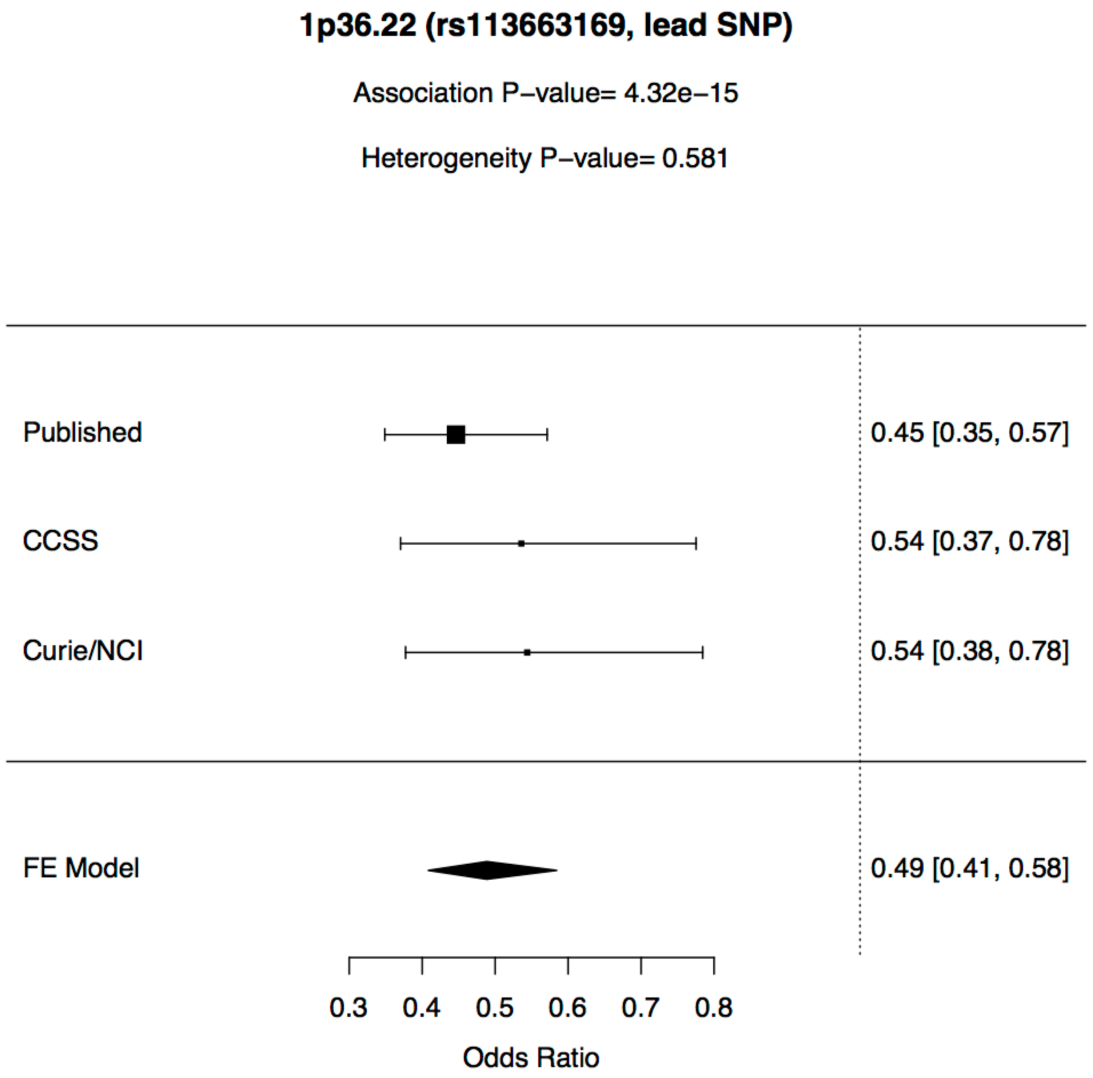

Supplementary Figure 2B. Meta-analysis forest plots for the 6p25.1 EWS susceptibility locus.

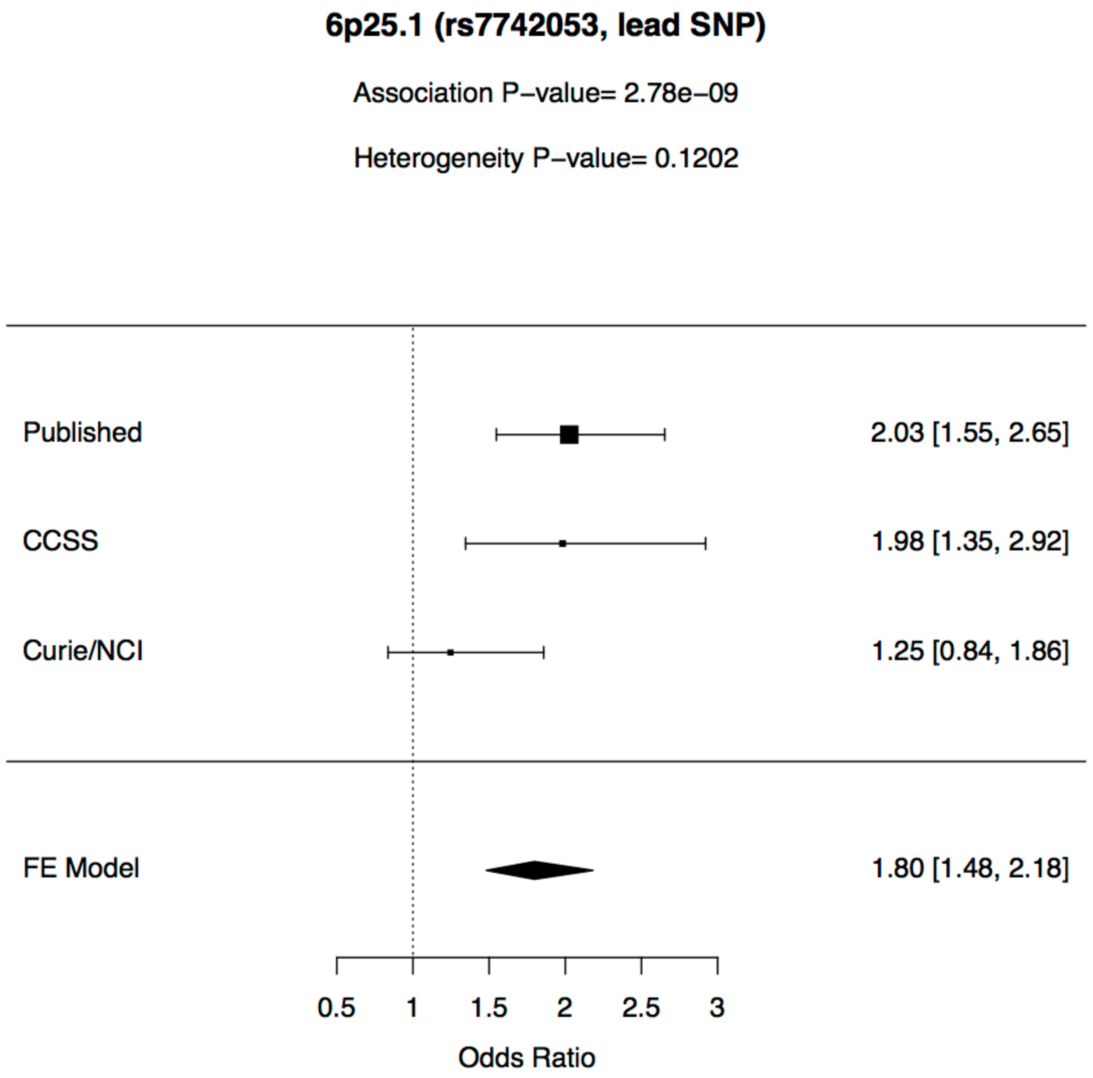

Supplementary Figure 2C. Meta-analysis forest plots for the 8q24.23 EWS susceptibility locus.

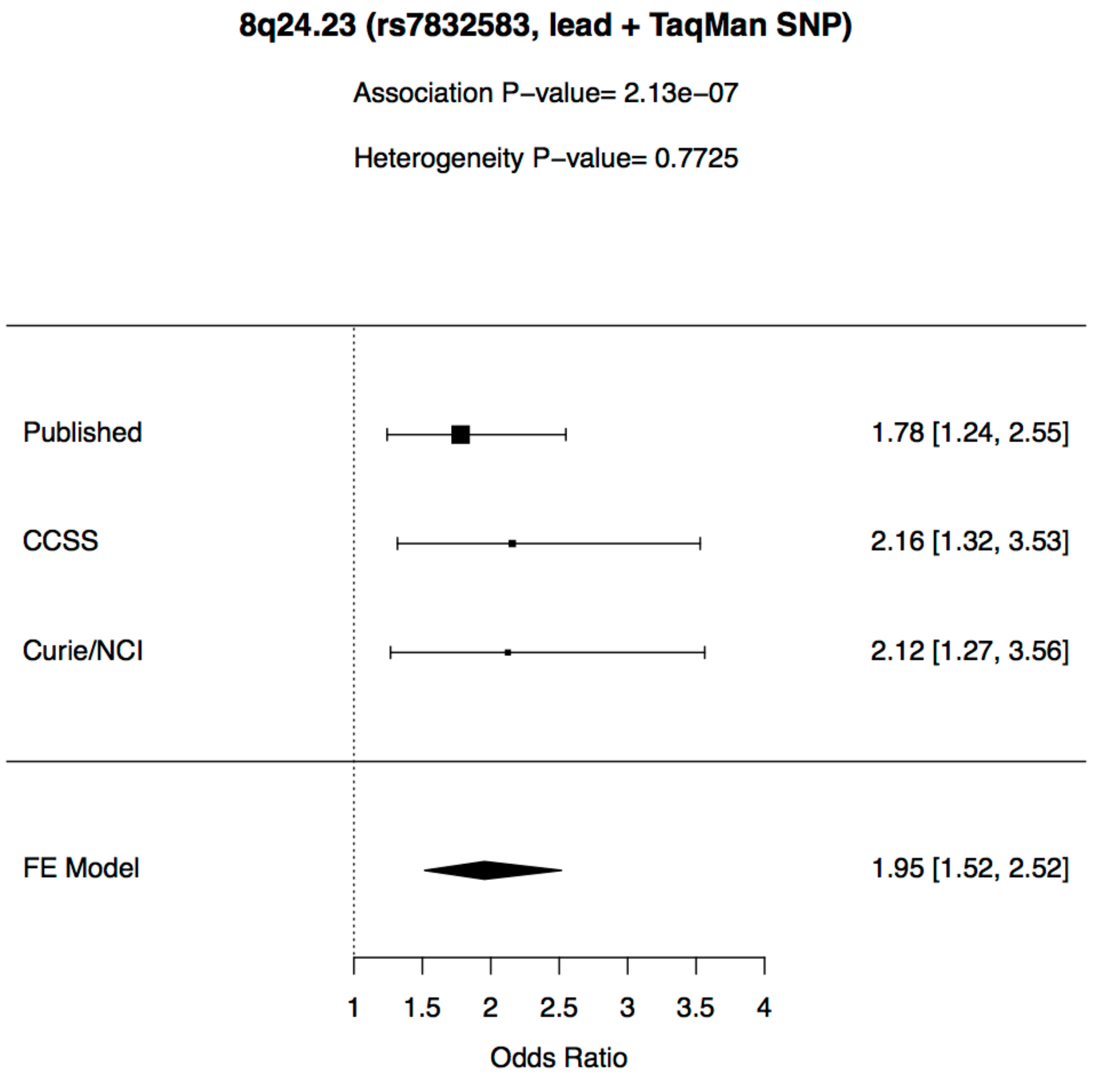

Supplementary Figure 2D. Meta-analysis forest plots for the 10q21.3 EWS susceptibility locus.

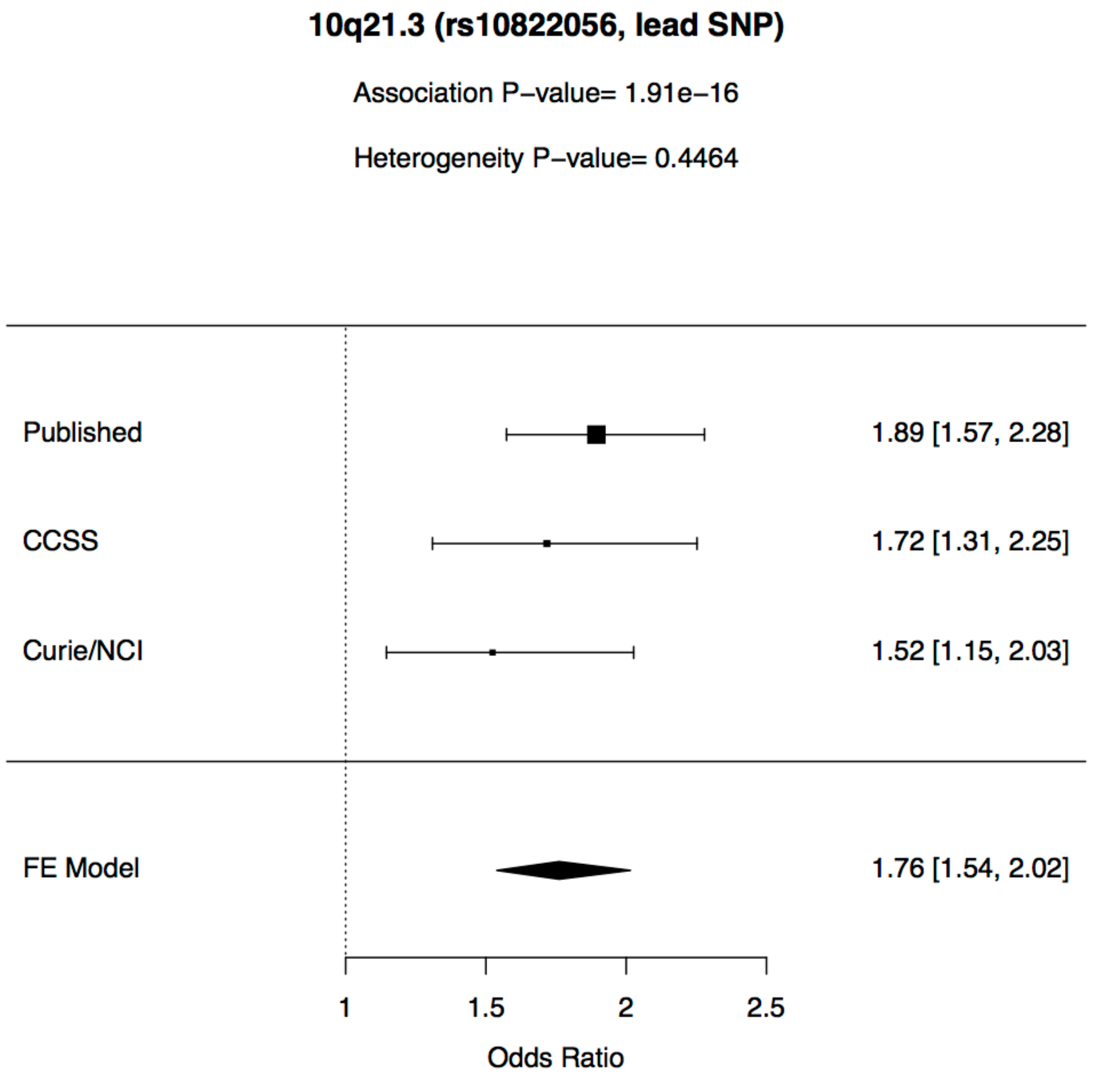

Supplementary Figure 2E. Meta-analysis forest plots for the 15q15.1 EWS susceptibility locus.

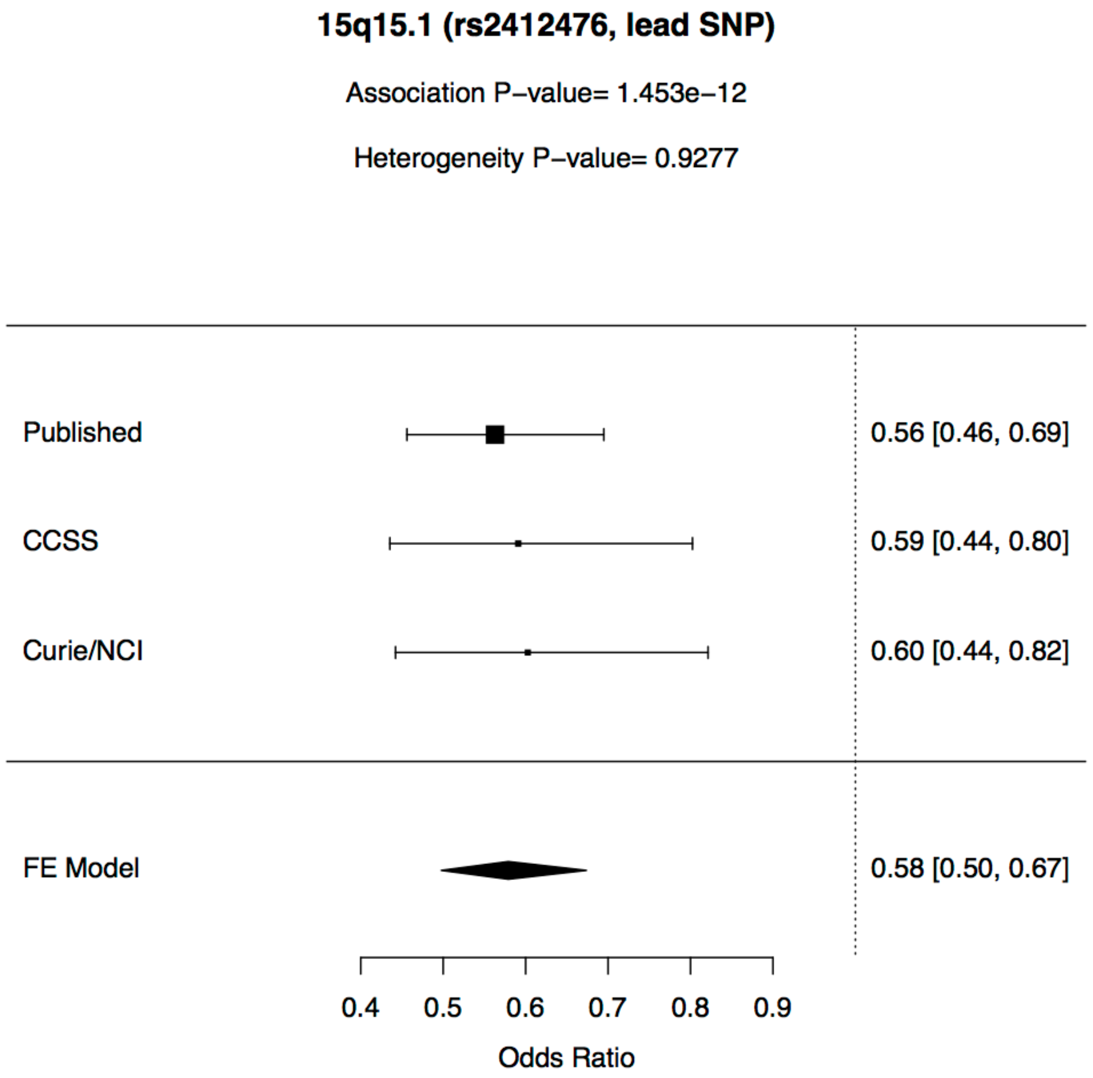

Supplementary Figure 2F. Meta-analysis forest plots for the 20p11.22 EWS susceptibility locus.

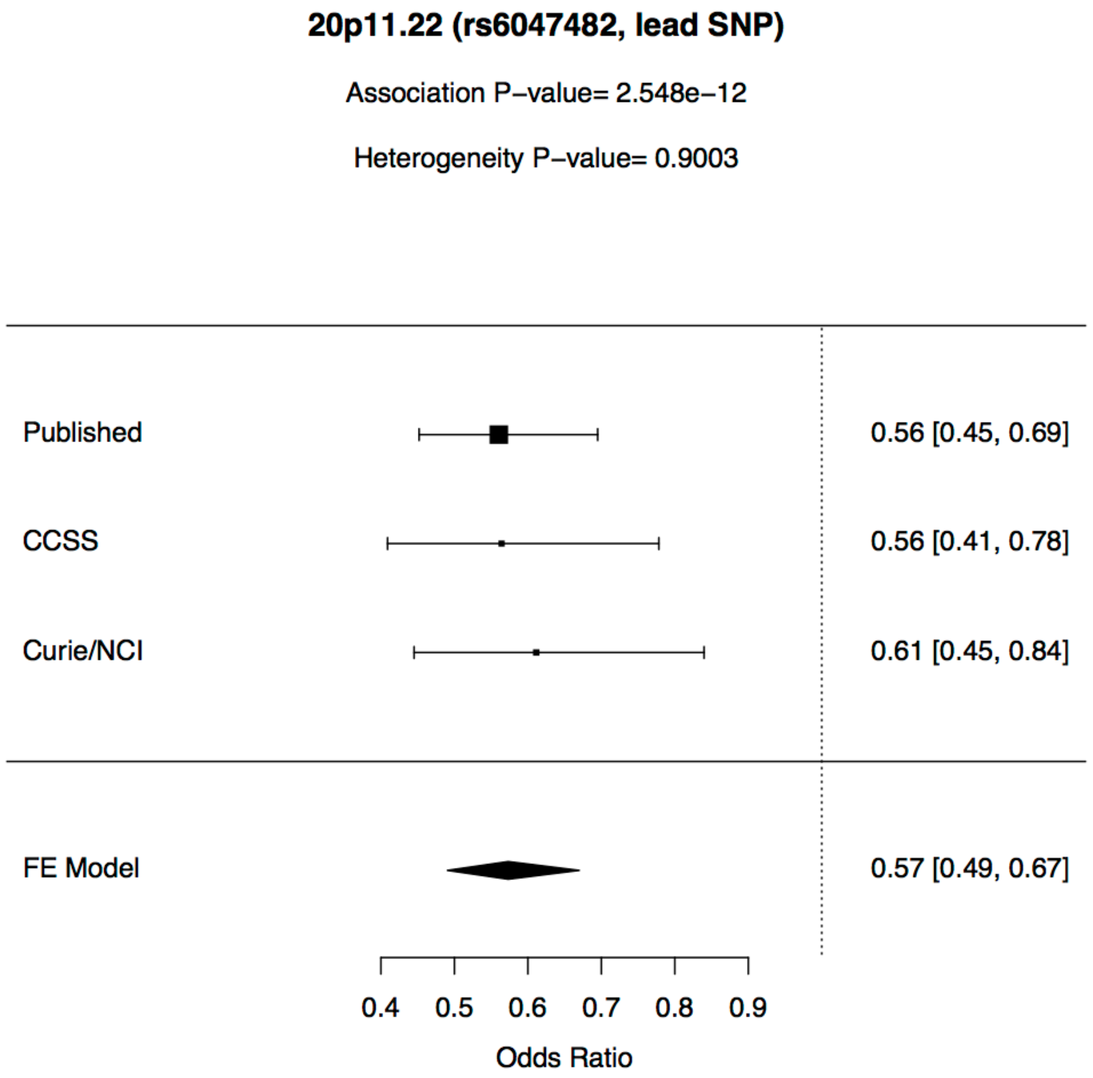

Supplementary Figure 2G. Meta-analysis forest plots for the 20p11.23 EWS susceptibility locus.

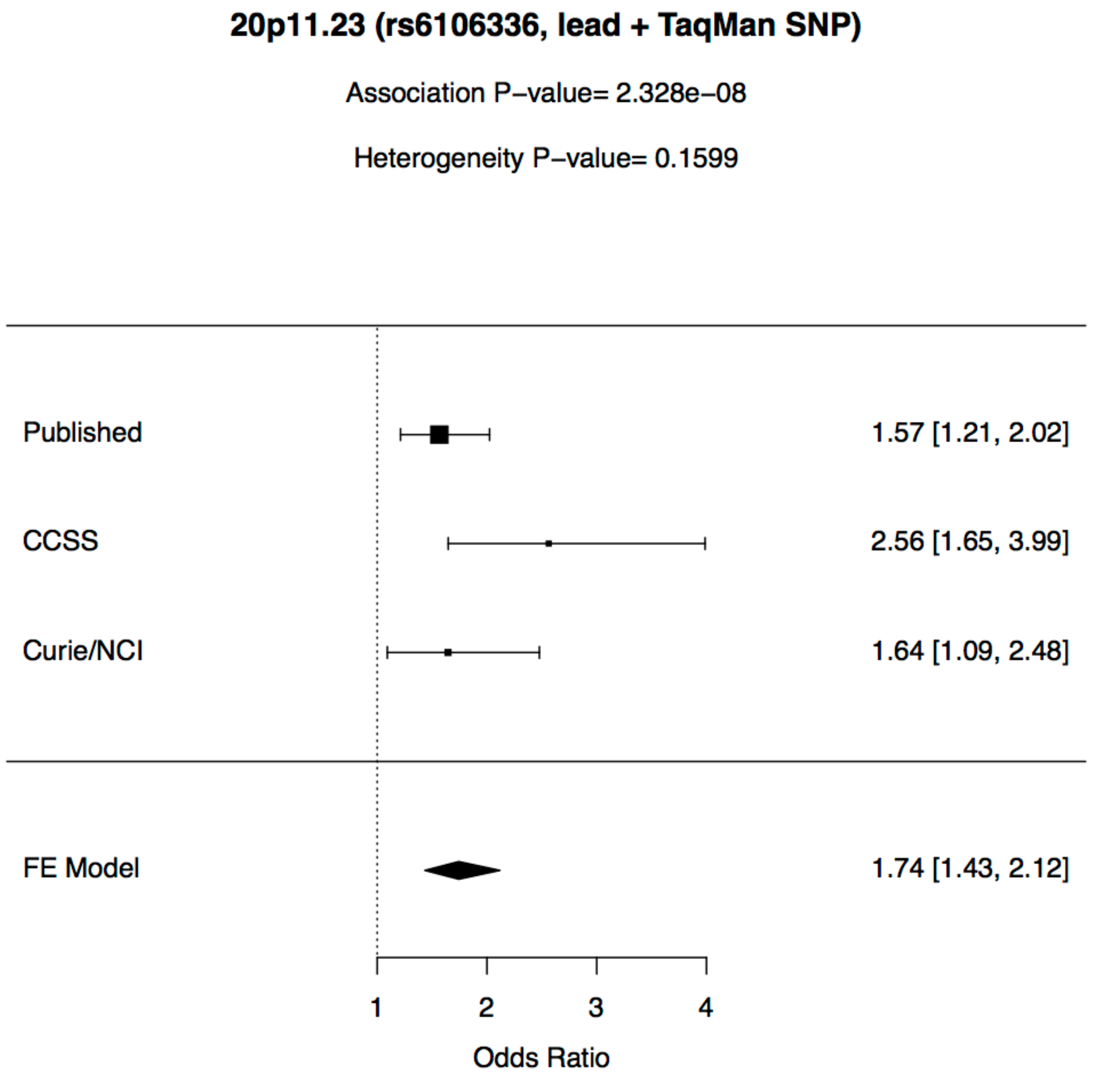

Supplementary Figure 3A. Local linkage disequilibrium and association P-value plot for variants in the 1p36.22 EWS susceptibility locus.

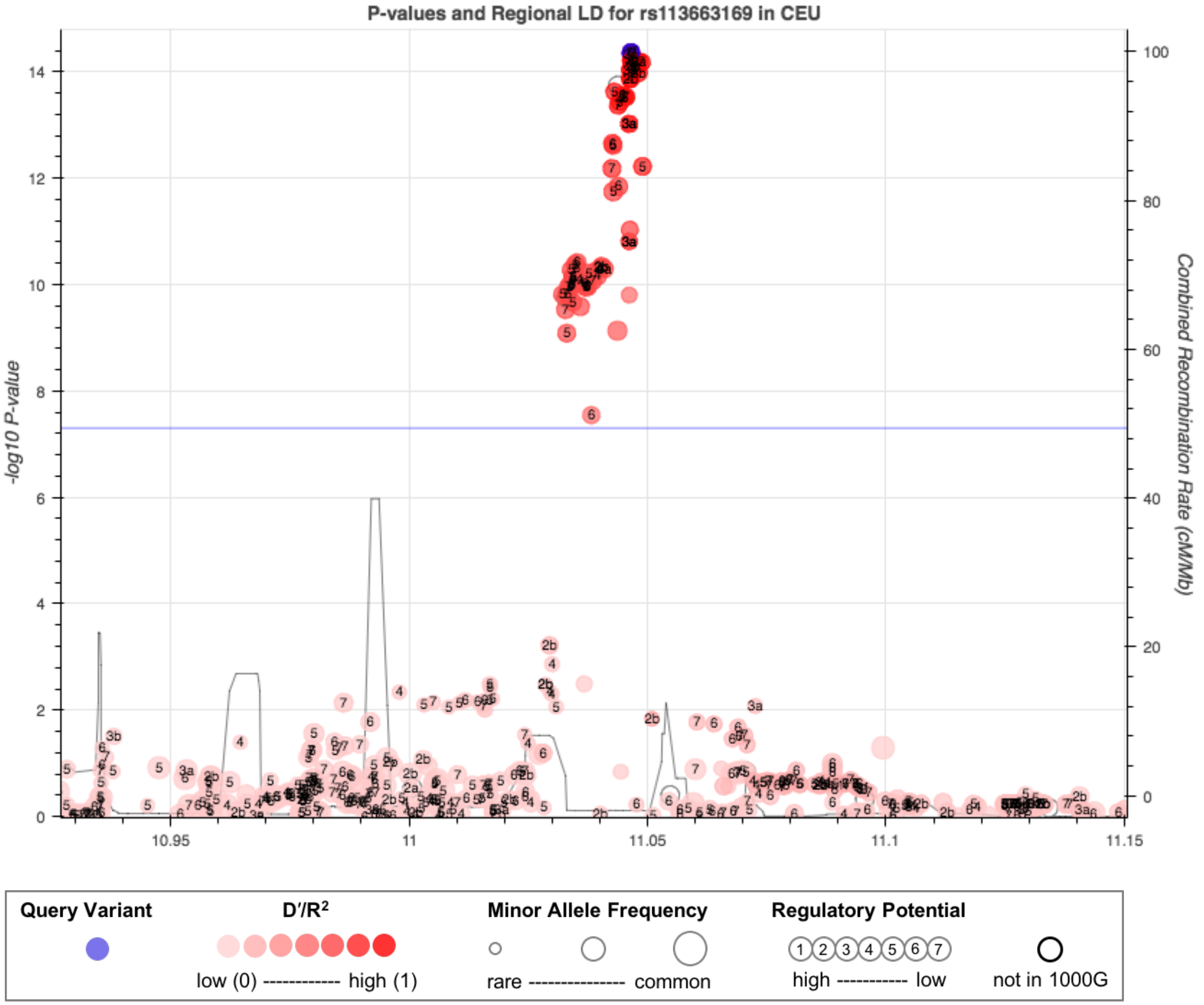

Supplementary Figure 3B. Local linkage disequilibrium and association P-value plot for variants in the 6p25.1 EWS susceptibility locus**.**

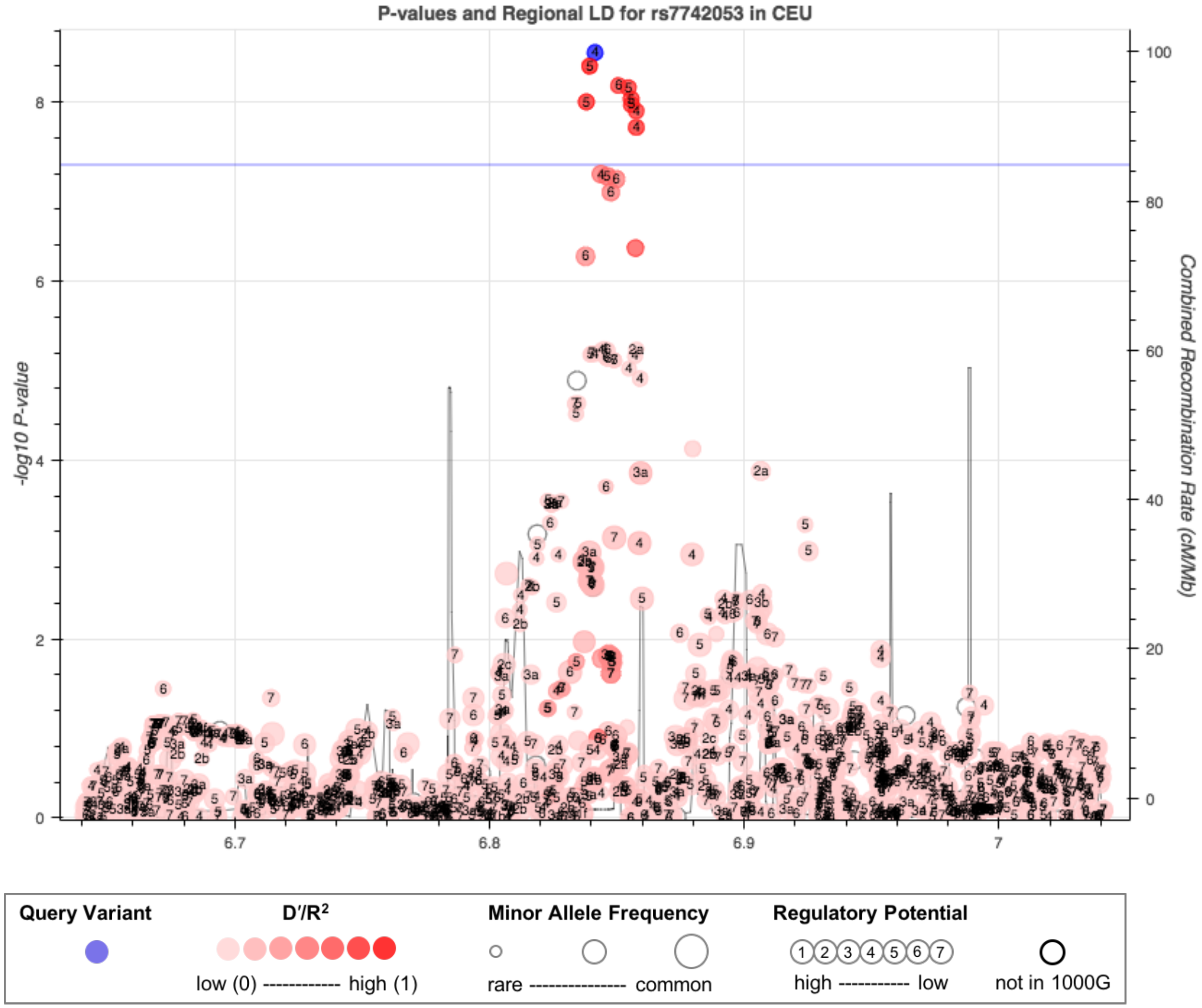

Supplementary Figure 3C. Local linkage disequilibrium and association P-value plot for variants in the 8q24.23 EWS susceptibility locus**.** The region is a gene desert with an uncharacterized long non-coding RNA, *LOC101927915*, and *FAM135B*, family with sequence similarity 135 member B mRNA, the closest transcripts. While the mutational landscape of EWS tumors is generally limited, chromosome 8 is observed to be recurrently gained in EWS tumors. It is notable that this 8q24.23 region is distinct from other established cancer susceptibility regions at 8q24.21.

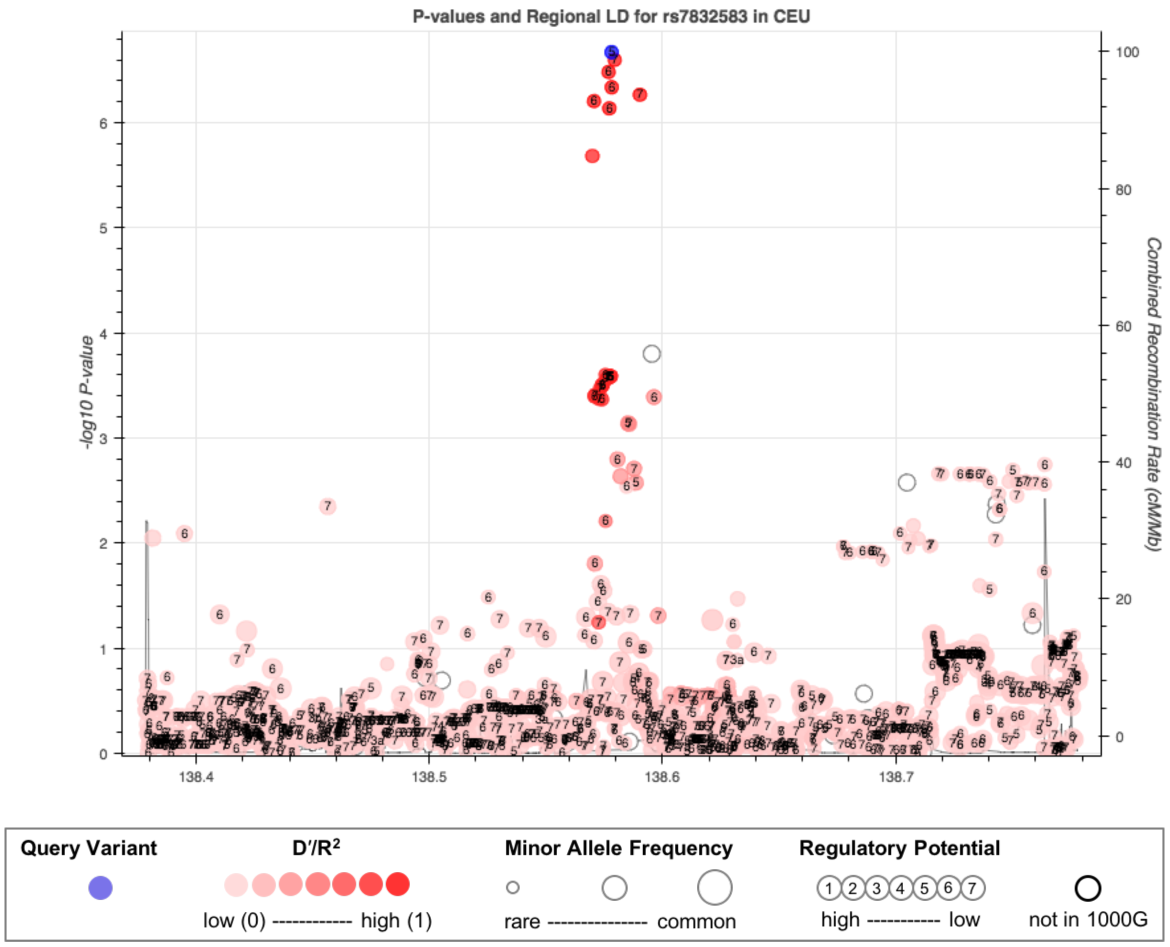

Supplementary Figure 3D. Local linkage disequilibrium and association P-value plot for variants in the 10q21.3 EWS susceptibility locus**.**

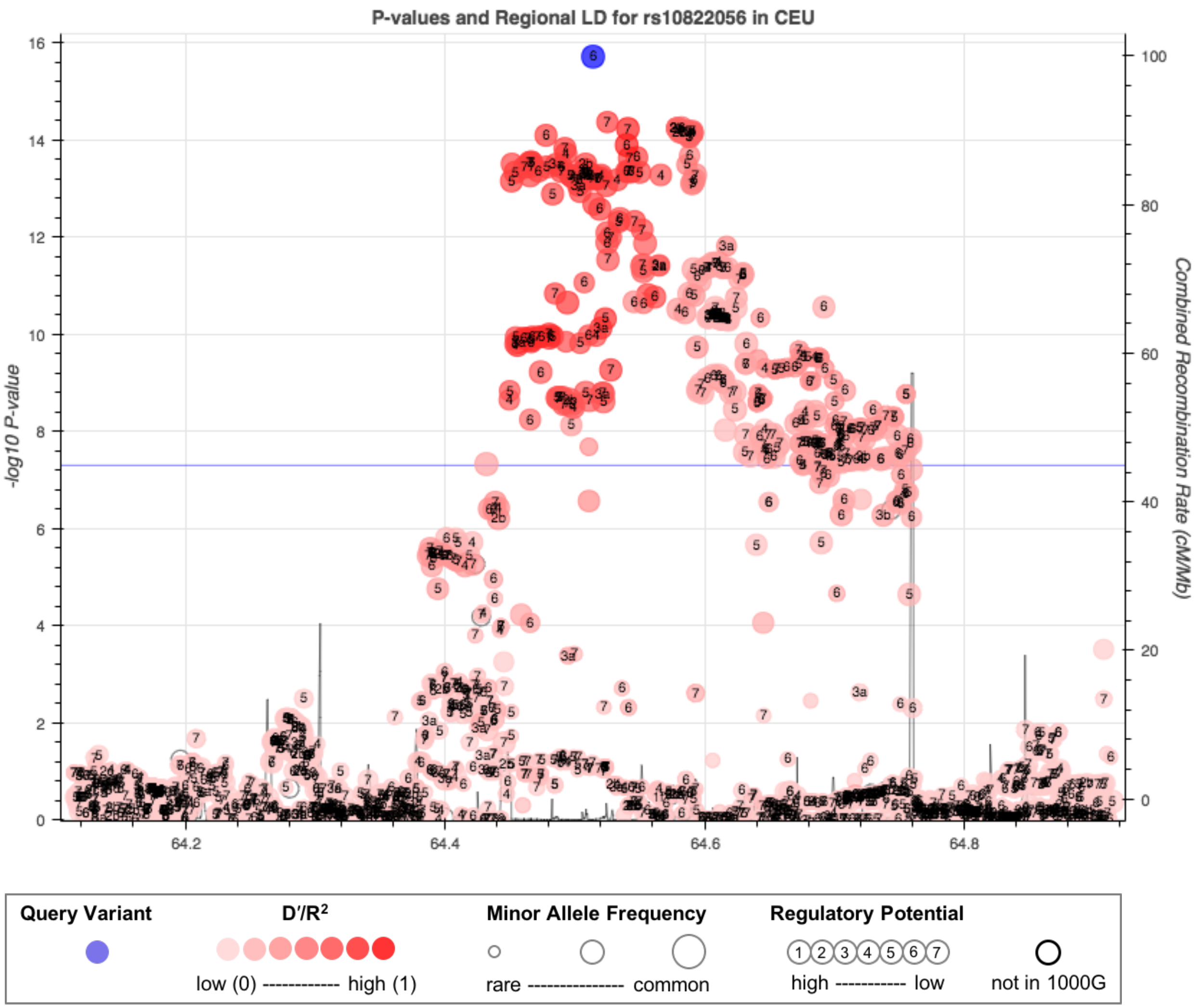

Supplementary Figure 3E. Local linkage disequilibrium and association P-value plot for variants in the 15q15.1 EWS susceptibility locus.

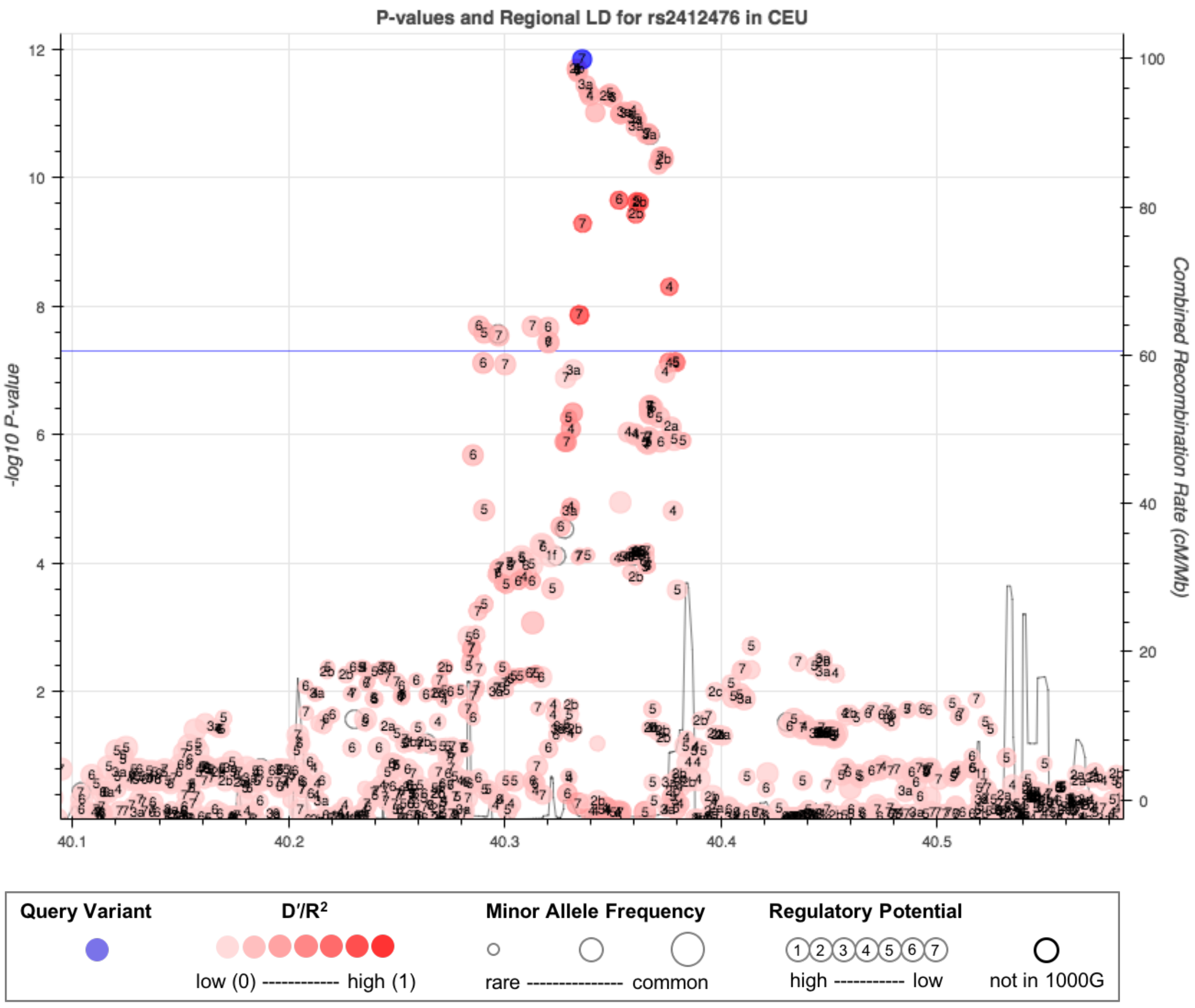

Supplementary Figure 3F. Local linkage disequilibrium and association P-value plot for variants in the 20p11.22 EWS susceptibility locus.

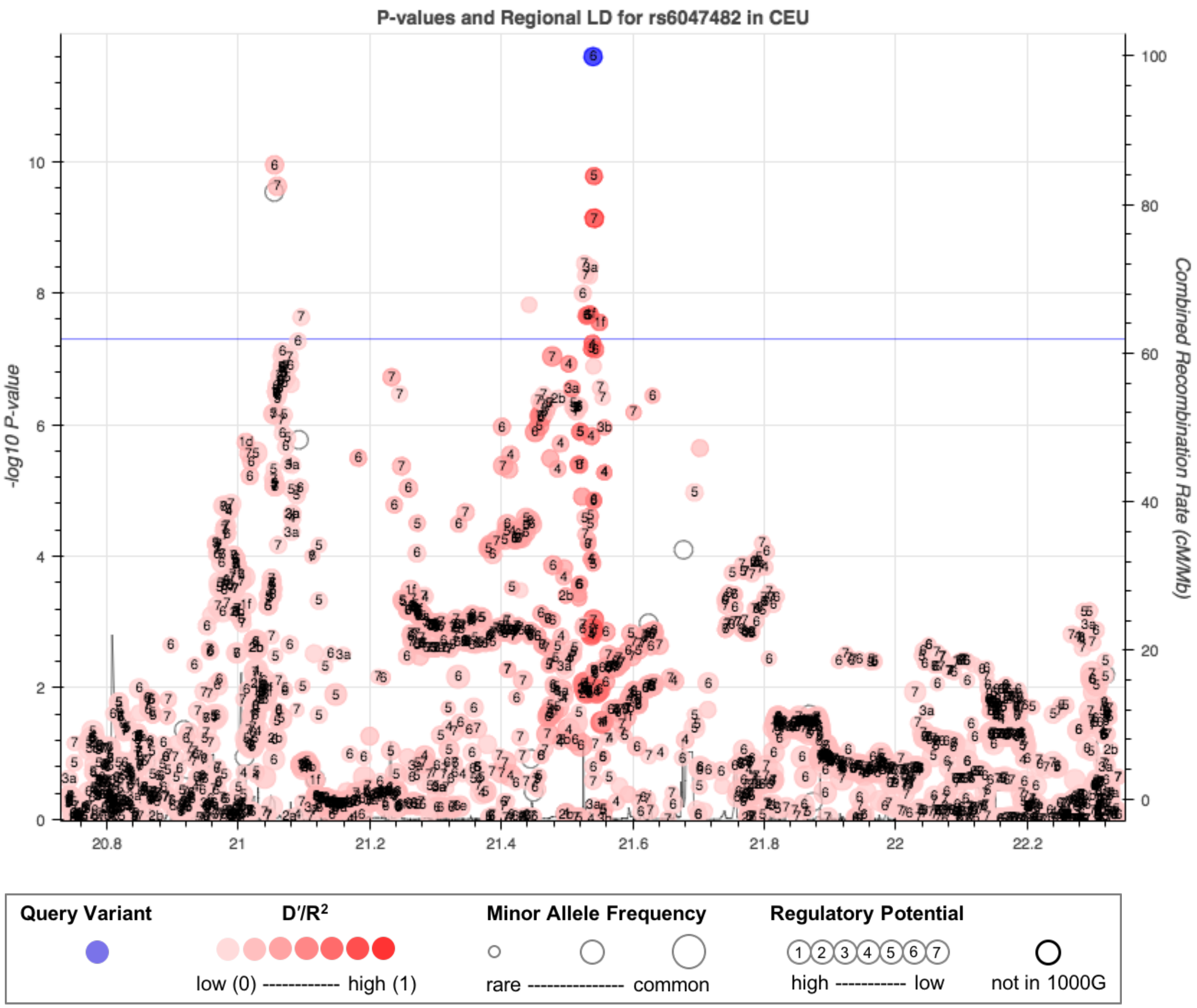

Supplementary Figure 3G. Local linkage disequilibrium and association P-value plot for variants in the 20p11.23 EWS susceptibility locus**.**

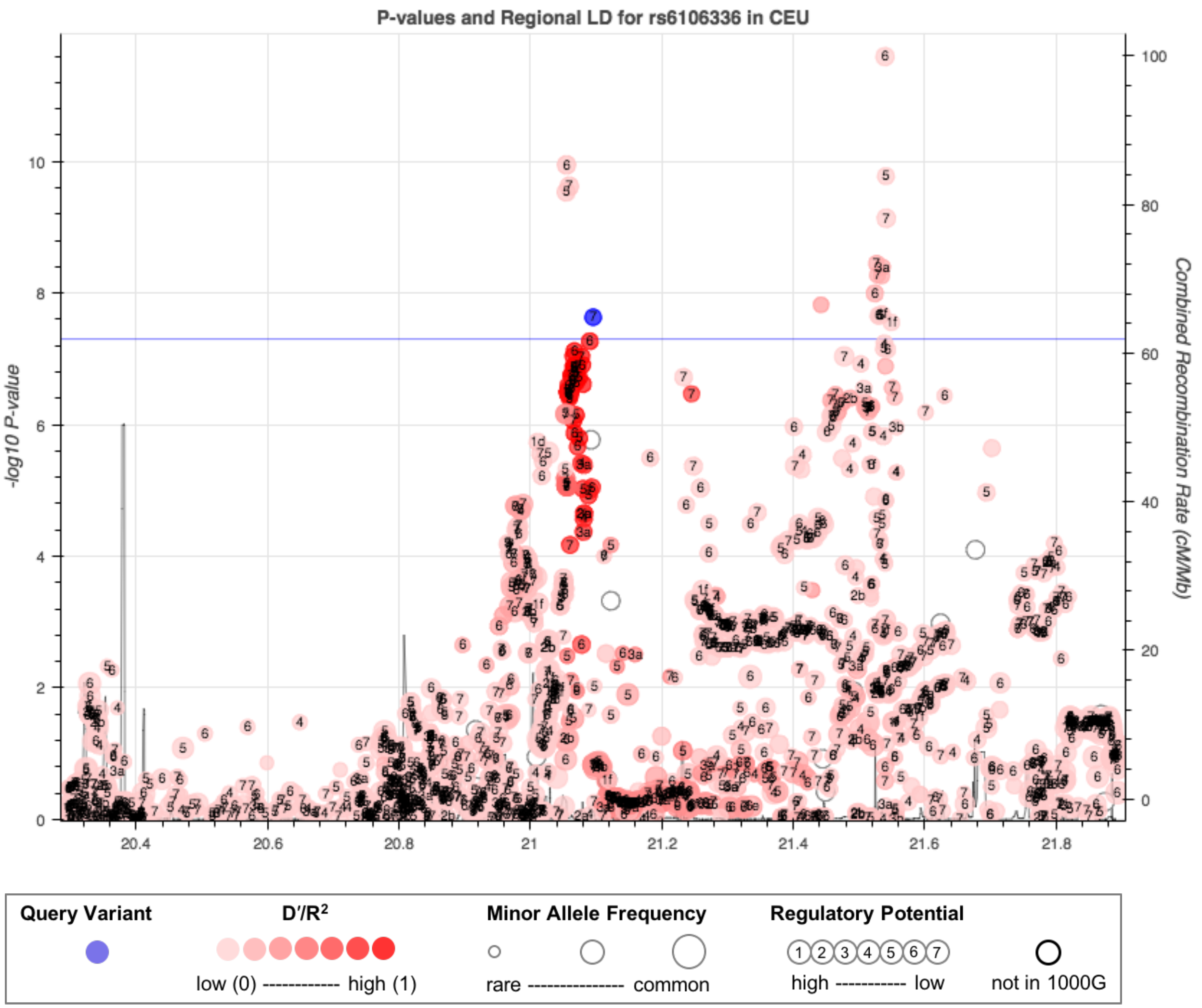

Supplementary Figure 4A. Plot of conditional analysis for the 1p36.22 EWS susceptibility region. Grey points are for the overall meta-analysis association and blue points are for the conditional analysis when conditioning on rs113663169.

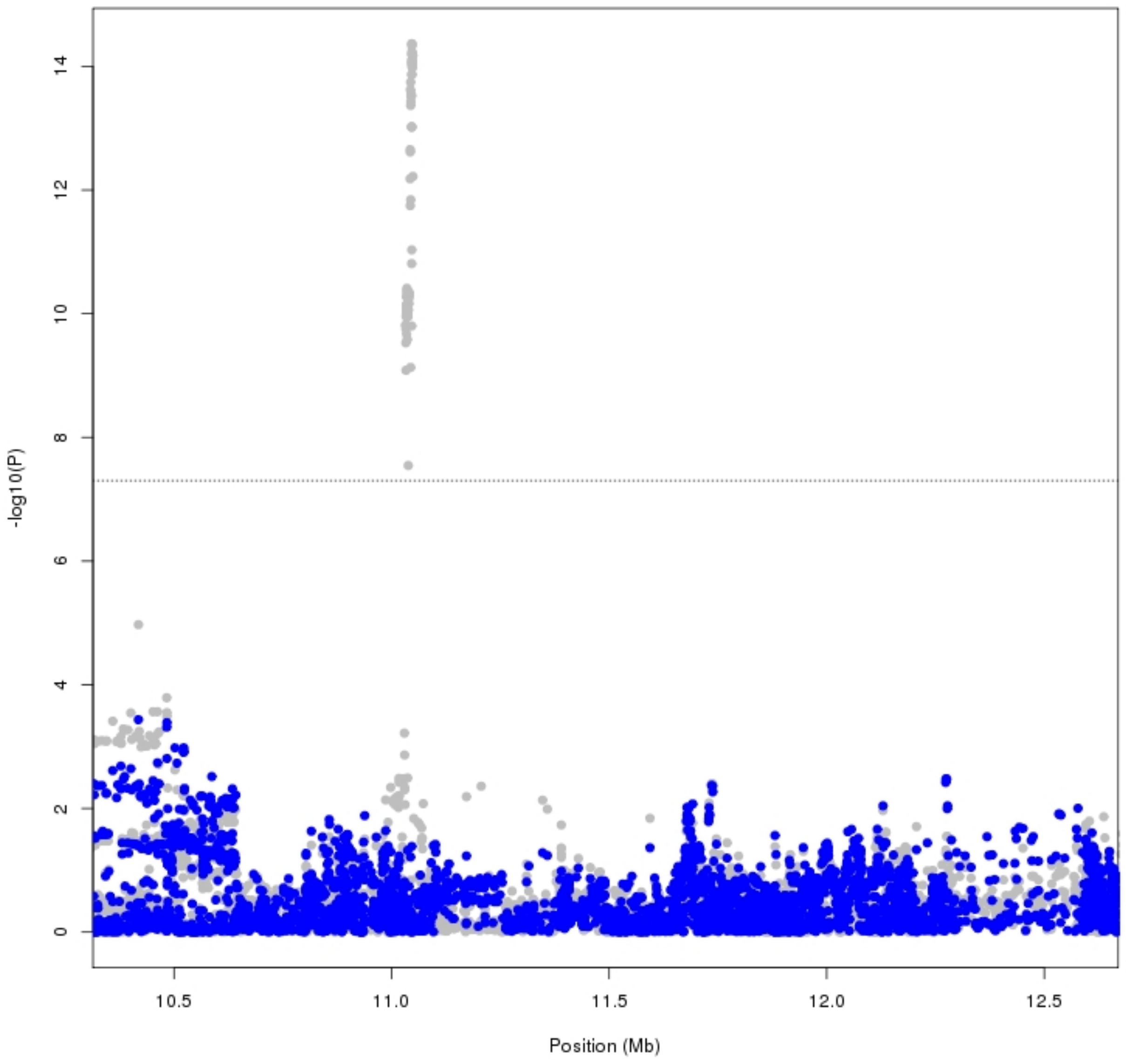

Supplementary Figure 4B. Plot of conditional analysis for the 6p25.1 EWS susceptibility region. Grey points are for the overall meta-analysis association and blue points are for the conditional analysis when conditioning on rs7742053.

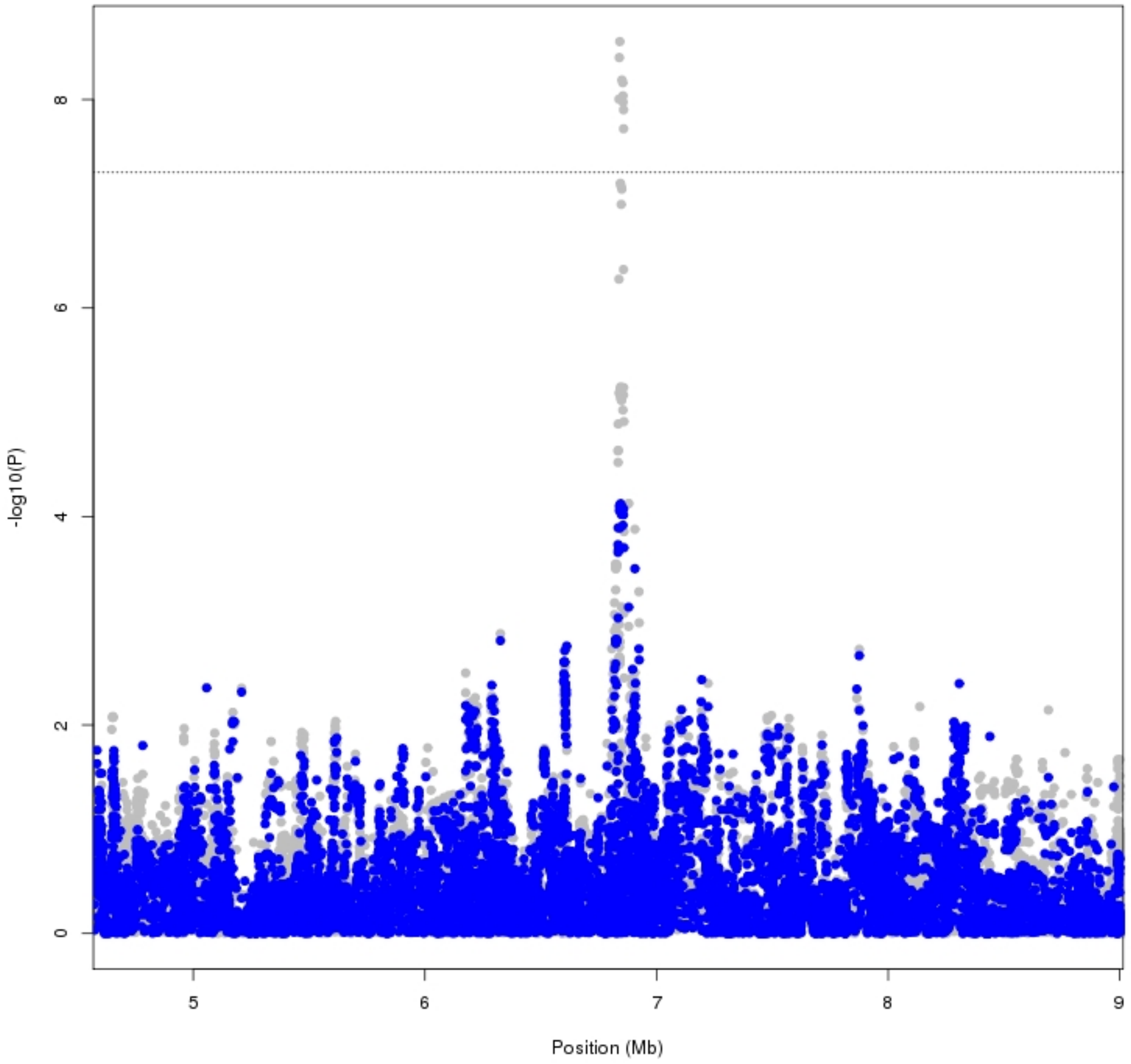

Supplementary Figure 4C. Plot of conditional analysis for the 8q24.23 EWS susceptibility region. Grey points are for the overall meta-analysis association and blue points are for the conditional analysis when conditioning on rs7832583.

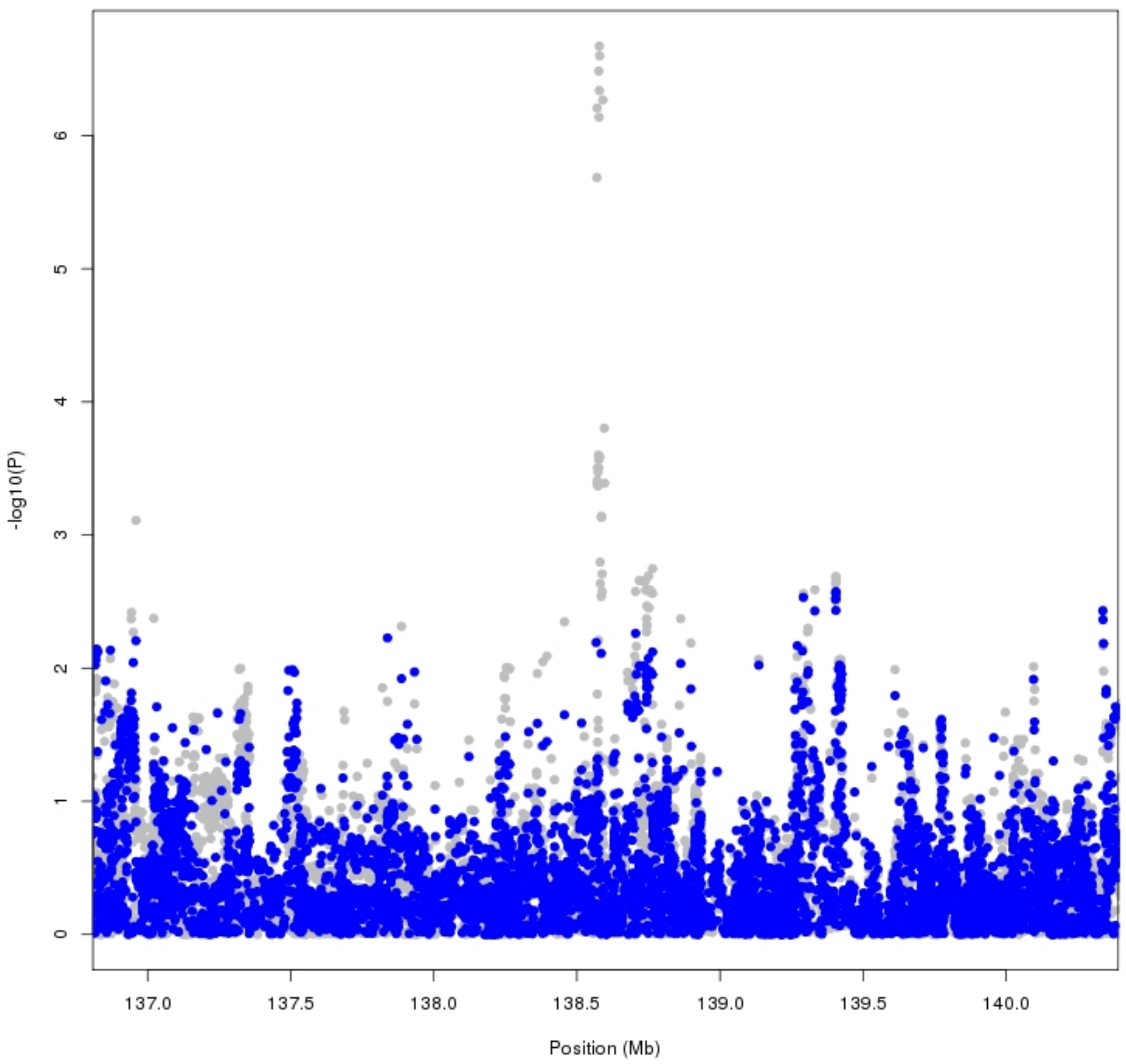

Supplementary Figure 4D. Plot of conditional analysis for the 10q21.3 EWS susceptibility region. Grey points are for the overall meta-analysis association and blue points are for the conditional analysis when conditioning on rs10822056.

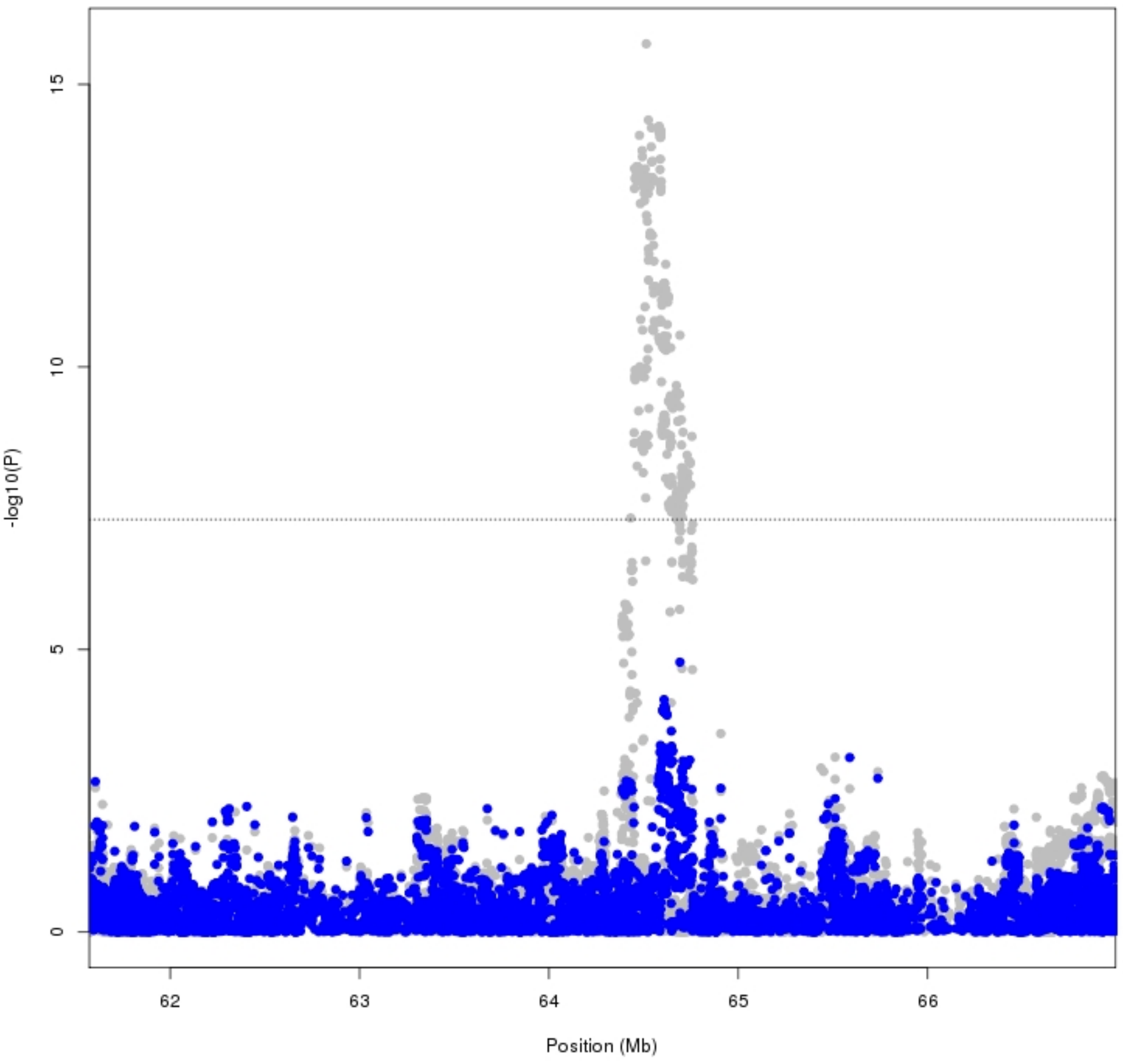

Supplementary Figure 4E. Plot of conditional analysis for the 15q15.1 EWS susceptibility region. Grey points are for the overall meta-analysis association and blue points are for the conditional analysis when conditioning on rs2412476.

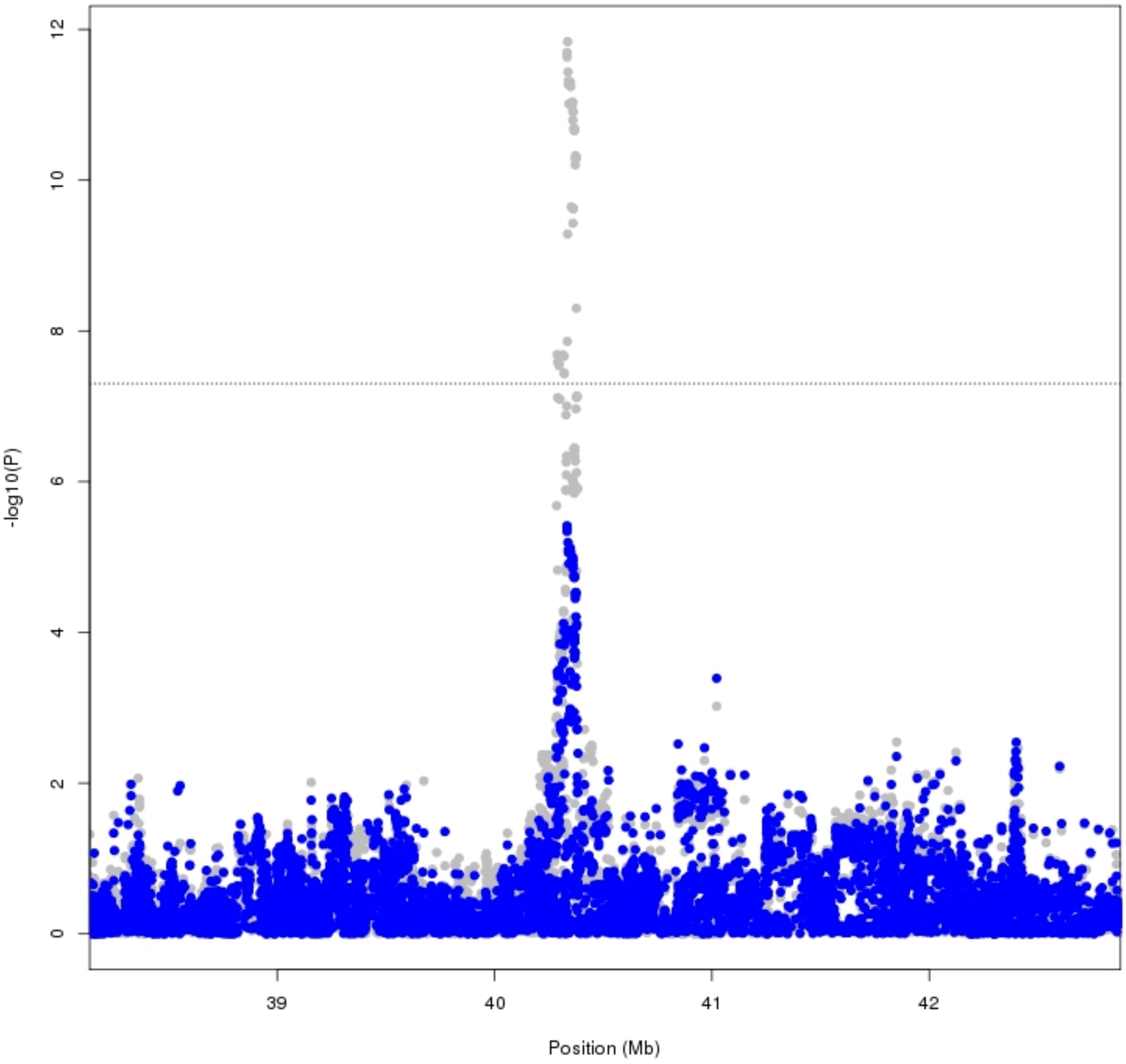

Supplementary Figure 4F. Plot of conditional analysis for the 20p11.22-23 EWS susceptibility region. Grey points are for the overall meta-analysis association, blue points are for the conditional analysis when conditioning on rs6047482 and red points are for the conditional analysis when conditioning on both rs6047482 and rs6106336.

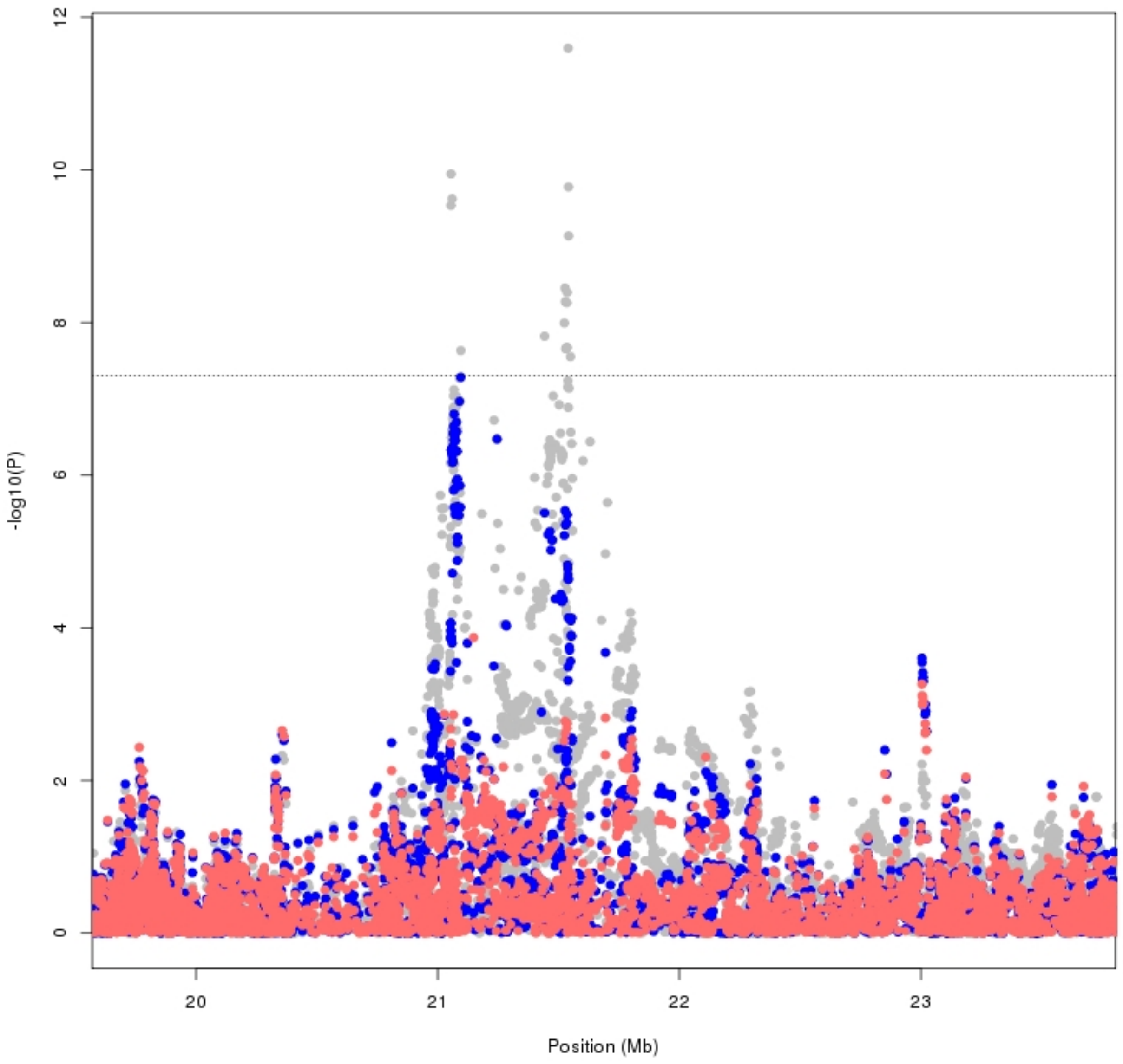

Supplementary Figure 5A. TaqMan replication of 6p25.1 locus (rs7744366) in independent European and German sets. The correlation with the 6p25.1 lead SNP (rs7742053) is an R^2 of 0.86.

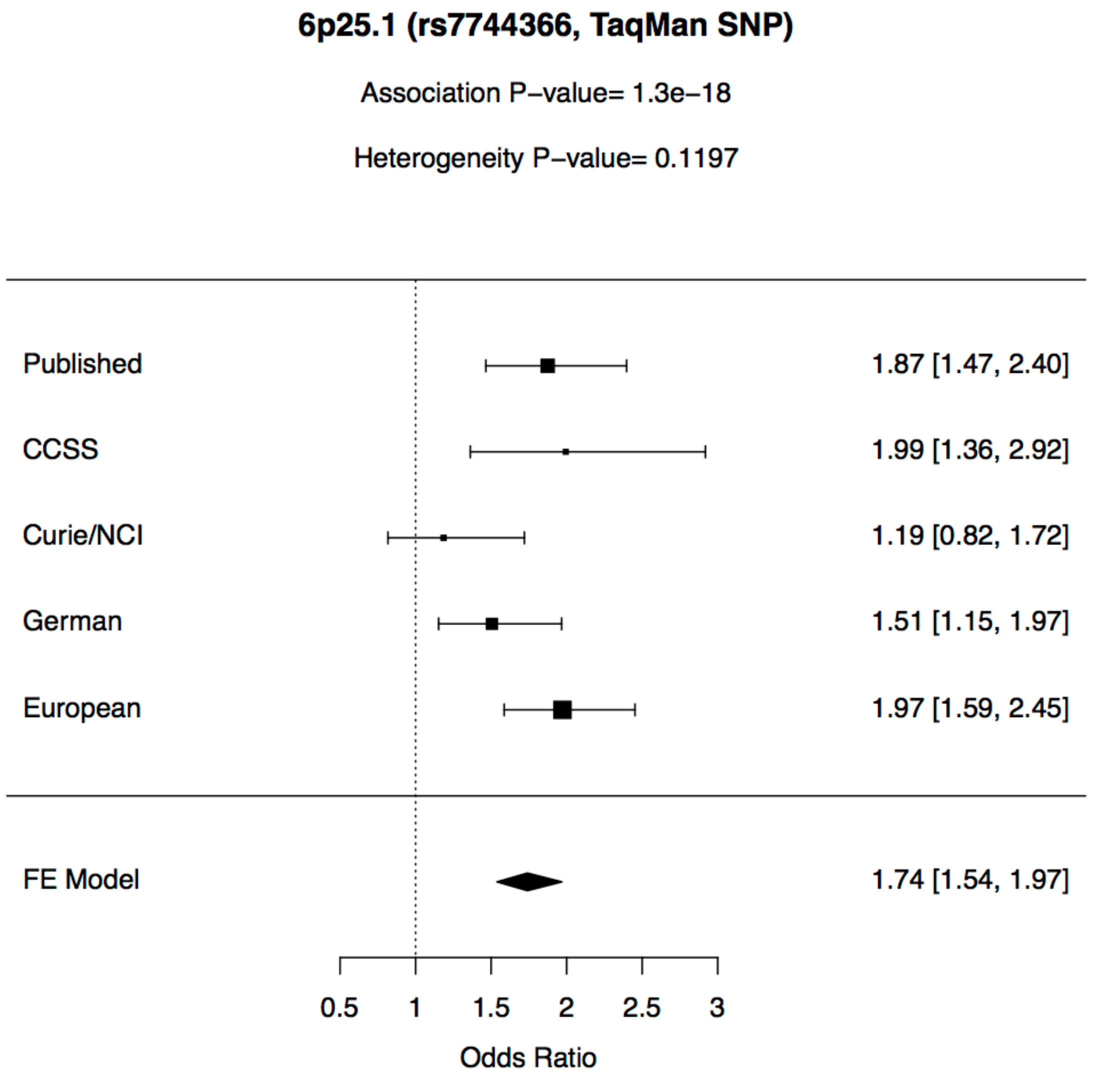

Supplementary Figure 5B. TaqMan replication of 8q24.23 locus (rs7832583) in independent European and German sets.

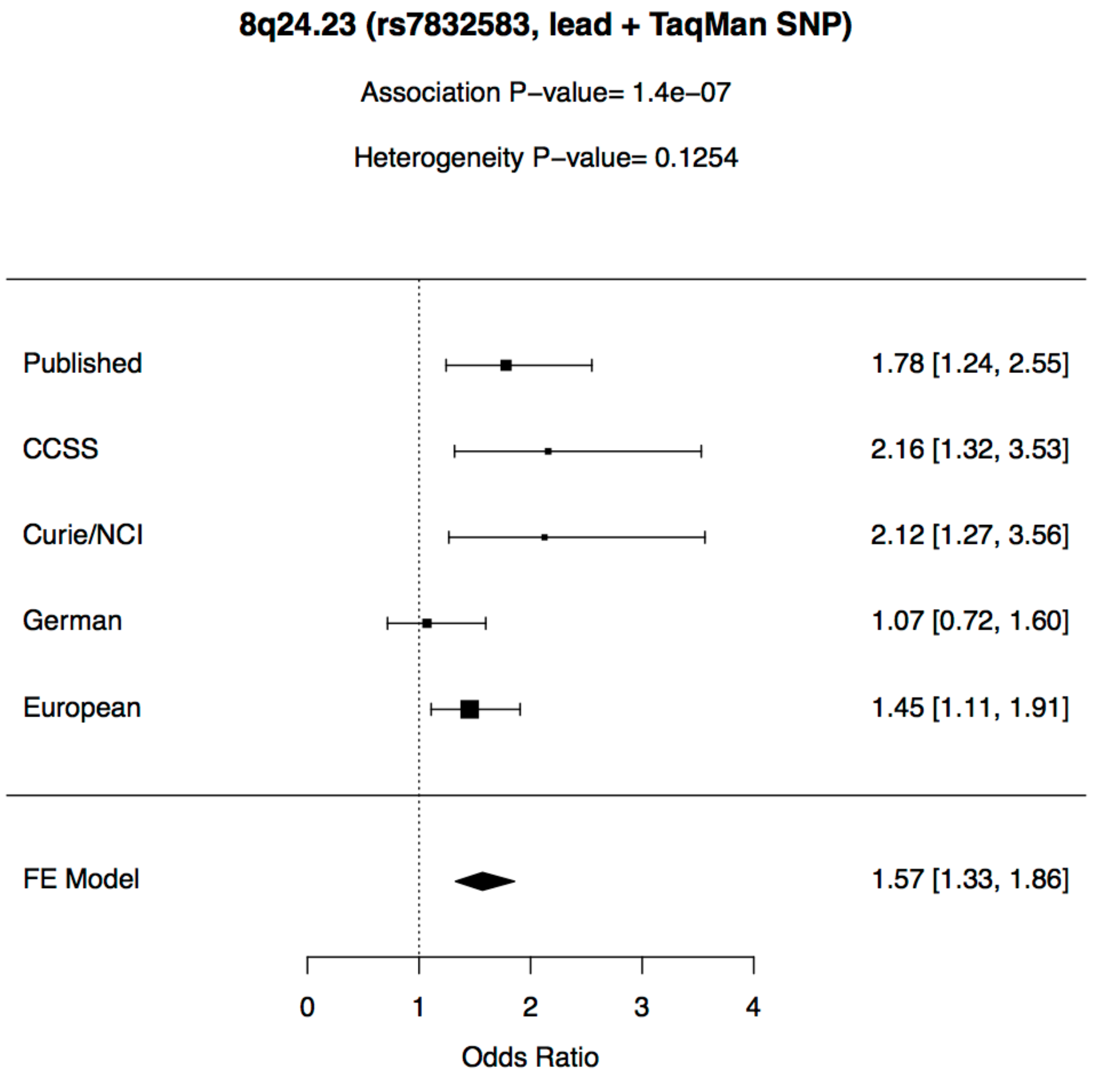

Supplementary Figure 5C. TaqMan replication of 20p11.22 locus (rs12106193) in independent European and German sets. The correlation with the 20p11.22 lead SNP (rs6047482) is an R^2 of 0.67.

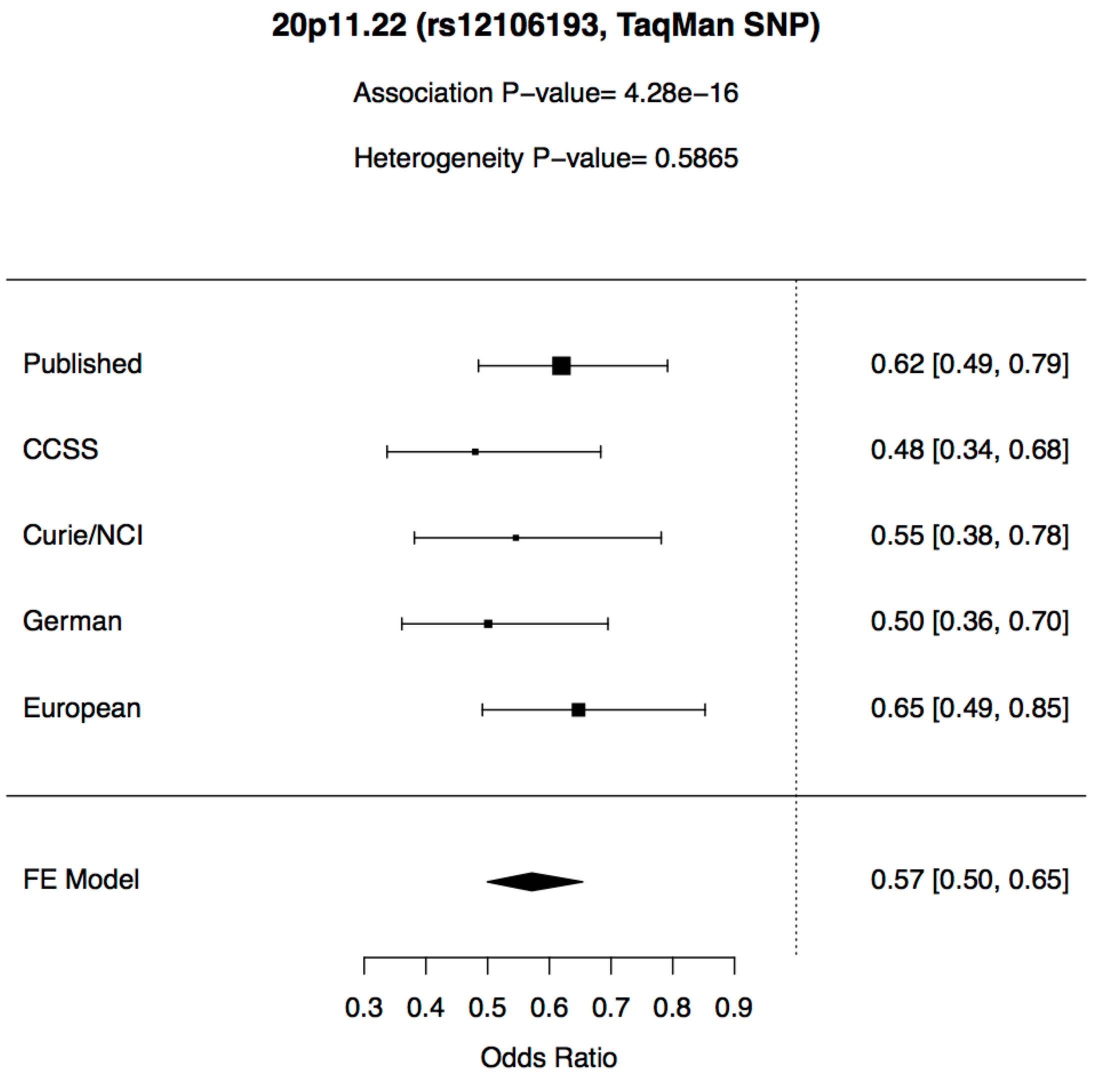

Supplementary Figure 5D. TaqMan replication of 20p11.23 locus (rs6106336) in independent European and German sets.

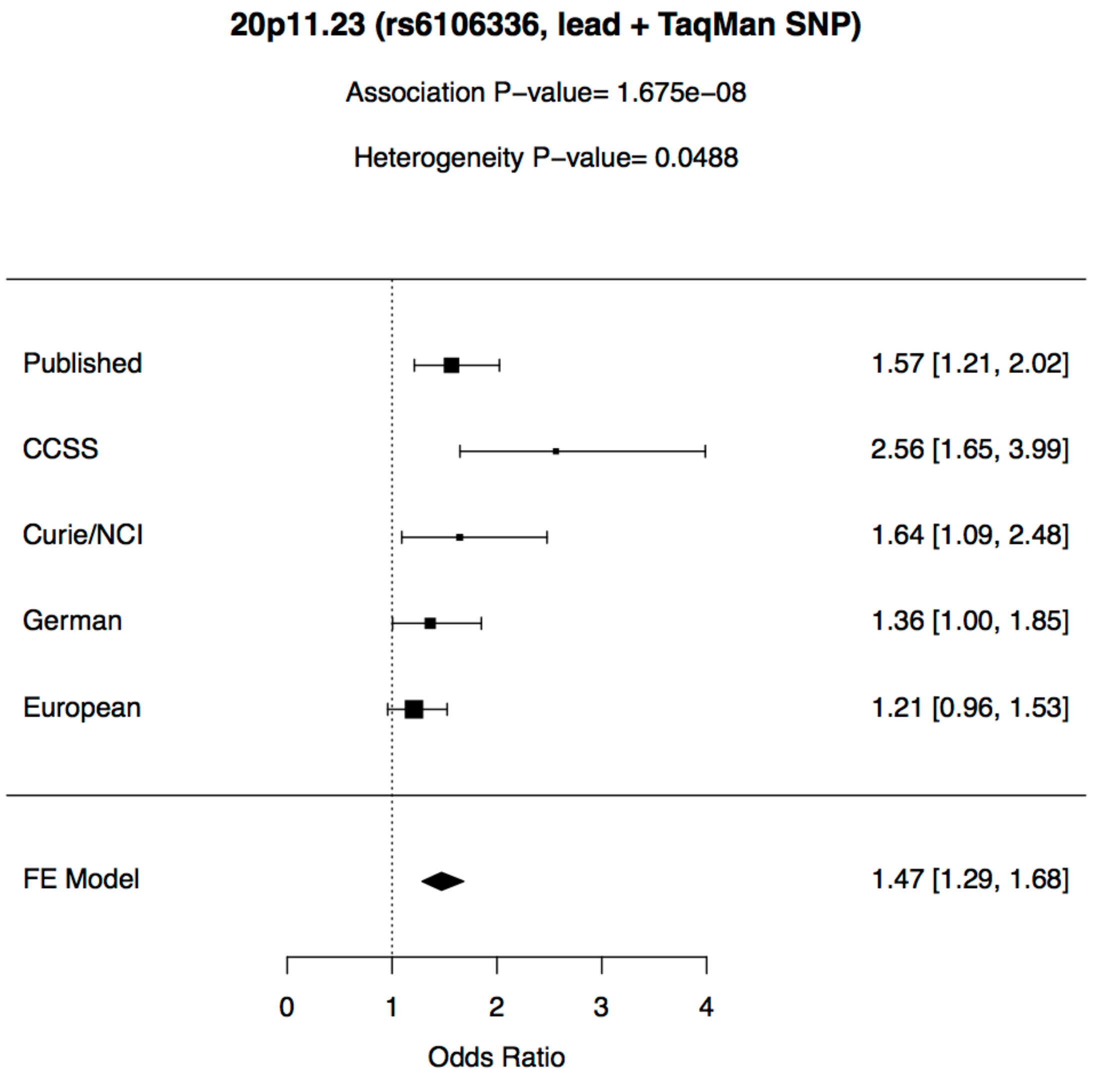

Supplementary Figure 6. Expression levels of *NKX2-2*, *KIZ* and *RREB1* in different sarcoma subtypes and pediatric solid tumors. Data are displayed as box-plots. Horizontals bars represent the medians, the boxes the interquartile range, and whiskers the 10th and 90th percentile, respectively. The number of analyzed samples per tumor entity is given in parentheses. ASPS, alveolar soft part sarcoma; ATRT, atypical-teratoid/rhabdoid-tumor; EW_LIKE, Ewing-like sarcoma; GIST, gastrointestinal stromal tumor; R-ARMS_pos, rhabdomyosarcoma alveolar fusion-positive; R-ARMS_neg, rhabdomyosarcoma alveolar fusion-negative; R-ERMS_neg, rhabdomyosarcoma embryonal fusion-negative; UNDIFF_sarcoma, undifferentiated sarcoma.

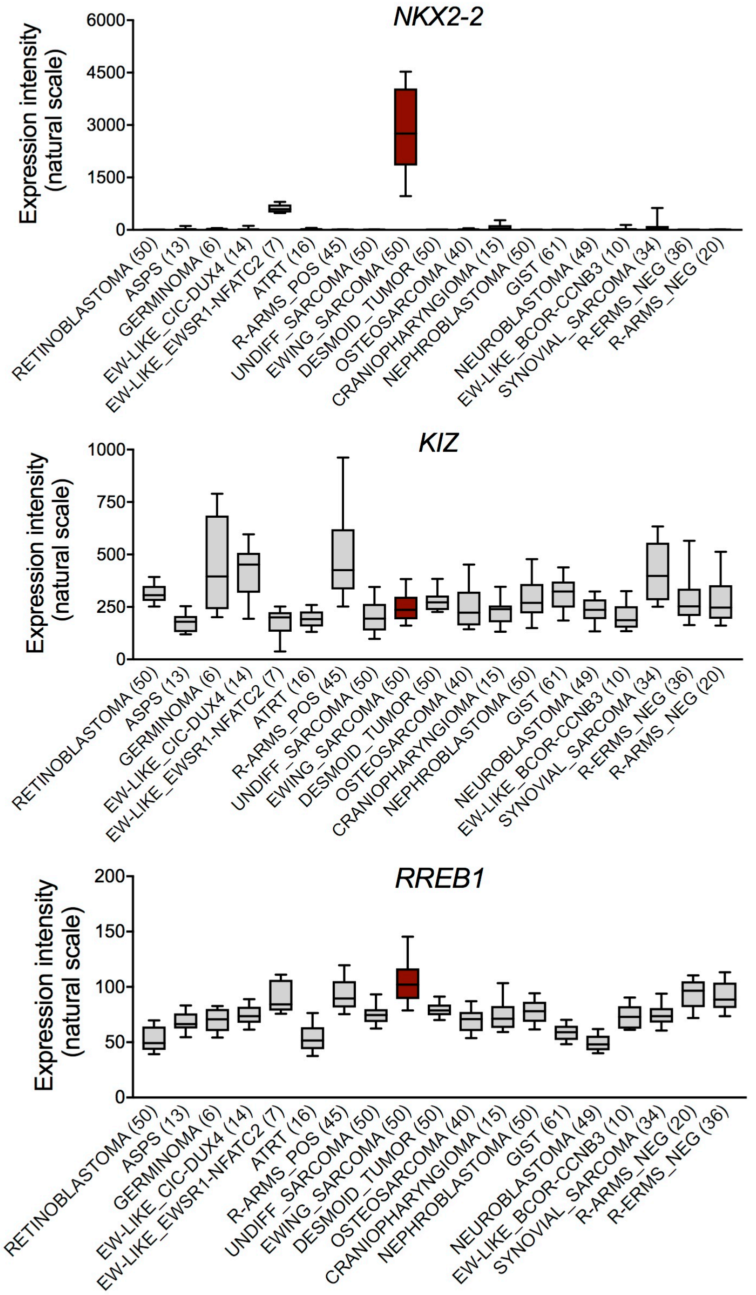

Supplementary Figure 7. Integrative view of lead SNP at 6p25.1 (rs7742053) and highly correlated CEU variants from LDlink, RefSeq genes, GGAA nucleotide repeat sequences, H3K27ac markers of open chromatin and FLI1 ChIP-seq for *EWSR1-FLI1* fusion binding in EWS cell lines A673 and TC71. A673 siEF1, A673 with siRNA-mediated knock down of *EWSR1-FLI1*.

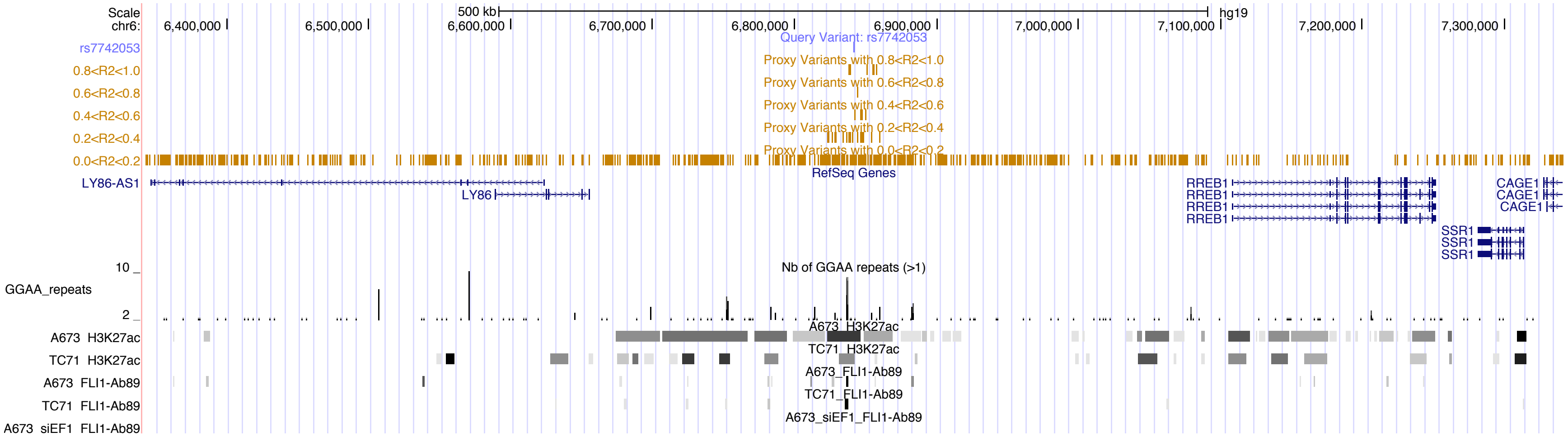

Supplementary Figure 8. Distance to EWSR1-FLI1 binding sites for the set of the top 6 EWS susceptibility variants and in a collection of 1,000 randomly selected SNPs from the same chromosomes in the EWS A673 and TC71 cell lines. There was a significantly closer distance to the nearest EWSR1-FLI1 binding site in EWS susceptibility variants (Wilcoxon P-value=0.0025 an 0.0009 in the A673 and TC71 cell lines, respectively), indicating enrichment of these binding sites near EWS susceptibility loci.

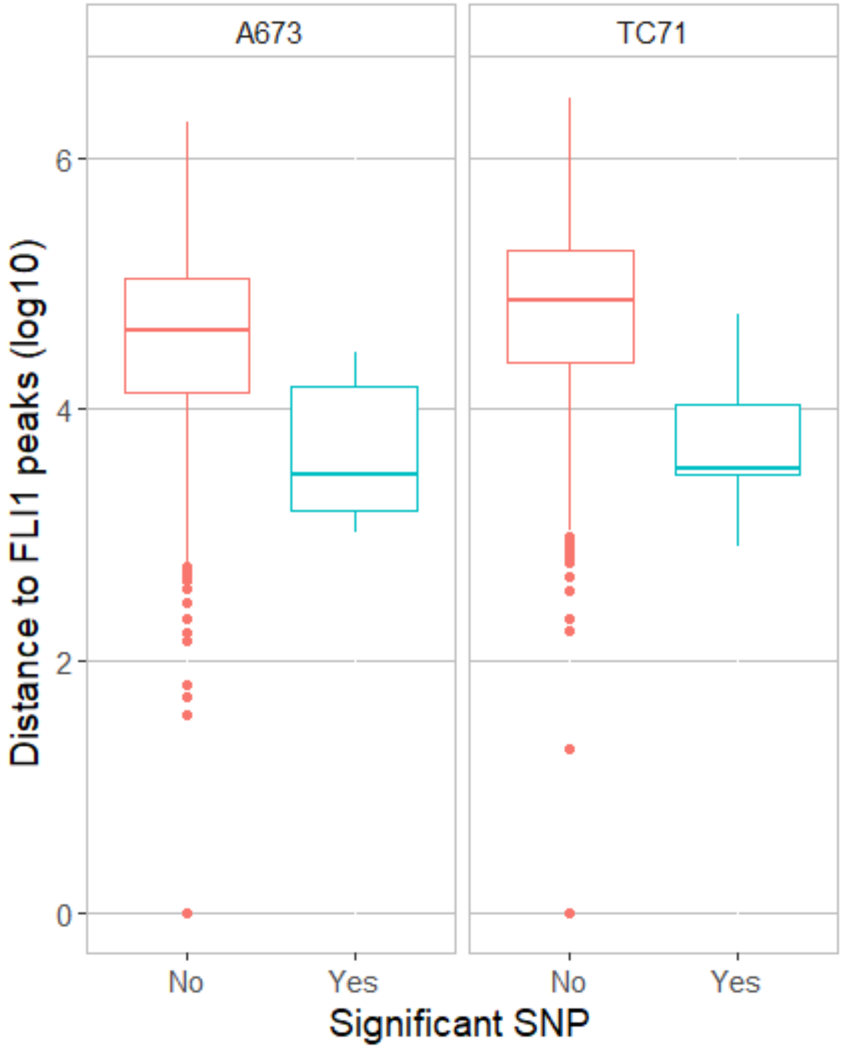

Supplementary Figure 9. *In vivo* fold change in gene expression of all coding genes and long non-coding RNAs at the 6p25.1 locus (brown), 8q24.23 locus (gray), and 20p11.22-23 locus (green) after doxycycline (Dox)-induced knock down of EWSR1-FLI1 in xenografts generated from A673/TR/shEF1 EWS cells grown in immunocompromised NSG mice.

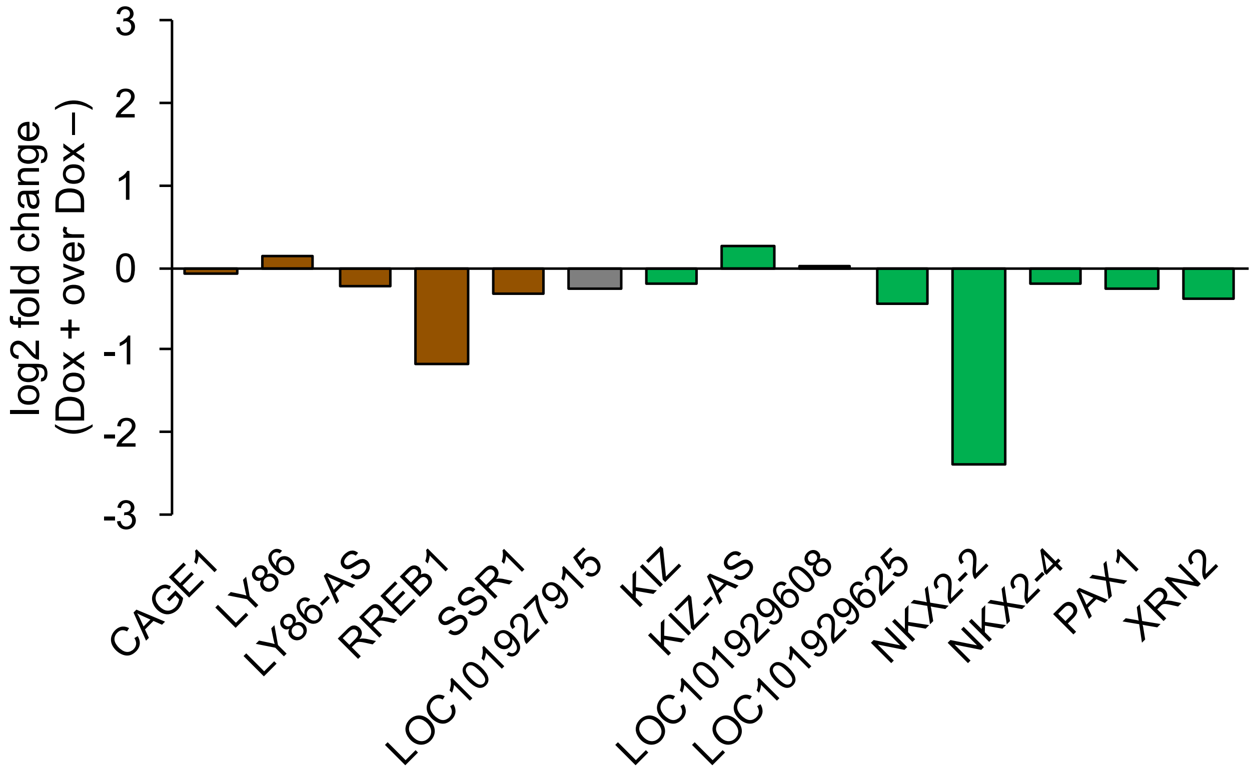

Supplementary Figure 10. Integrative view of lead SNP at 20p11.22 (rs6047482) and highly correlated CEU variants from LDlink, RefSeq genes, GGAA nucleotide repeat sequences, H3K27ac markers of open chromatin and FLI1 ChIP-seq for *EWSR1-FLI1* fusion binding in EWS cell lines A673 and TC71. A673 siEF1, A673 with siRNA-mediated knock down of *EWSR1-FLI1.*

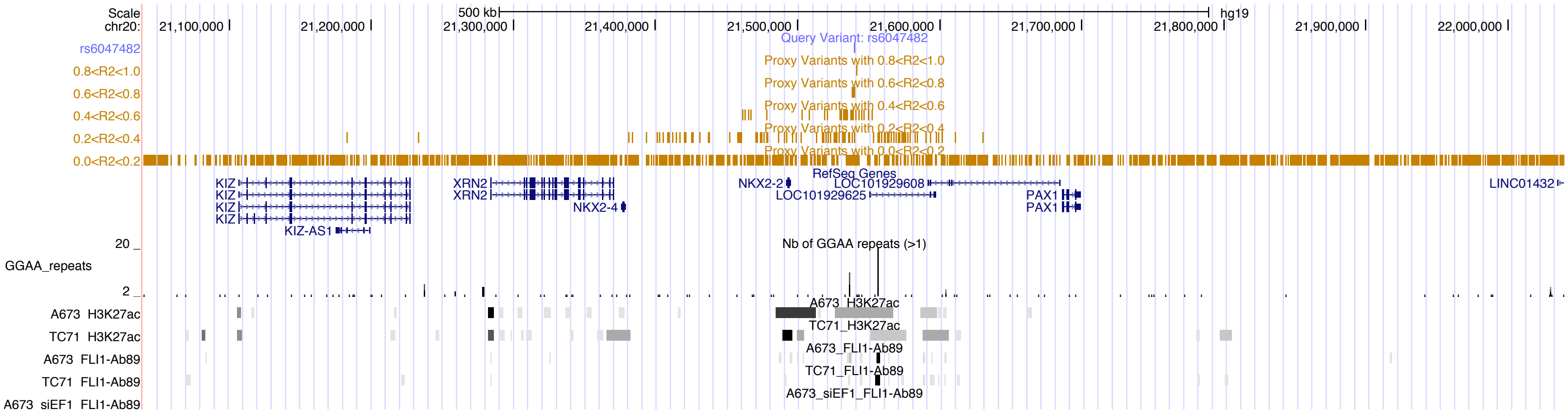

Supplementary Figure 11. Integrative view of lead SNP at 20p11.23 (rs67425864) and highly correlated CEU variants from LDlink, RefSeq genes, GGAA nucleotide repeat sequences, H3K27ac markers of open chromatin and FLI1 ChIP-seq for *EWSR1-FLI1* fusion binding in EWS cell lines A673 and TC71. A673 siEF1, A673 with siRNA-mediated knock down of *EWSR1-FLI1.*

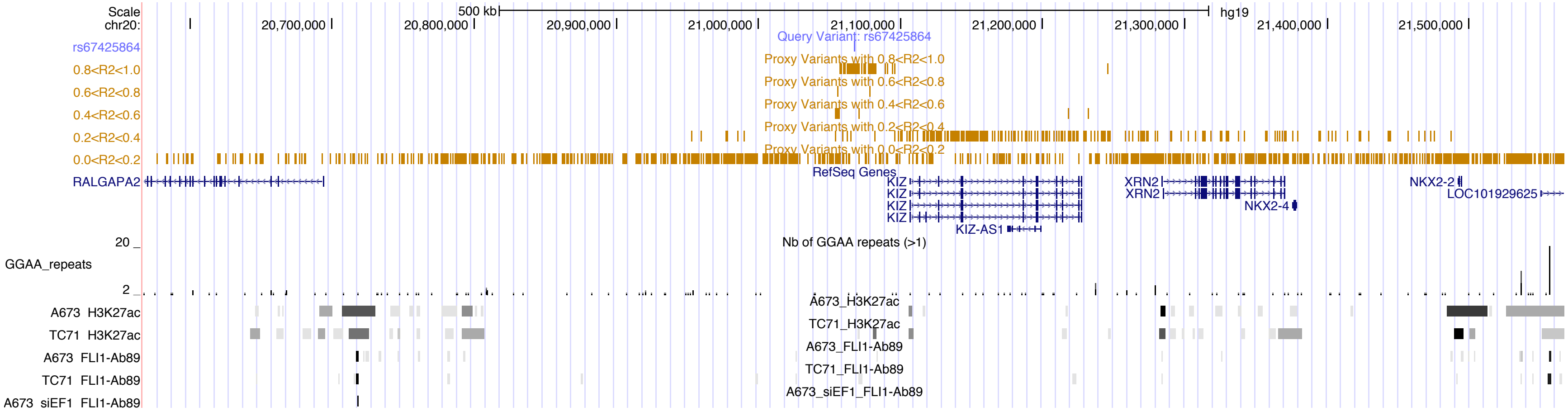

Supplementary Figure 12. EWS genetic risk score (GRS) combining genotype data for the 6 EWS susceptibility loci. (**A**) The distribution comparing cancer-free adult controls (blue) to EWS cases (red). On average, EWS cases carry 1.08 more risk alleles at EWS susceptibility loci than controls (7.08 average risk alleles in EWS cases, 6.01 average risk alleles in controls; P-value=2.44×10^{-63}). (**B**) Area under the receiver operating characteristic curve (AUC=0.7149) comparing the EWS GRS in cases and controls.

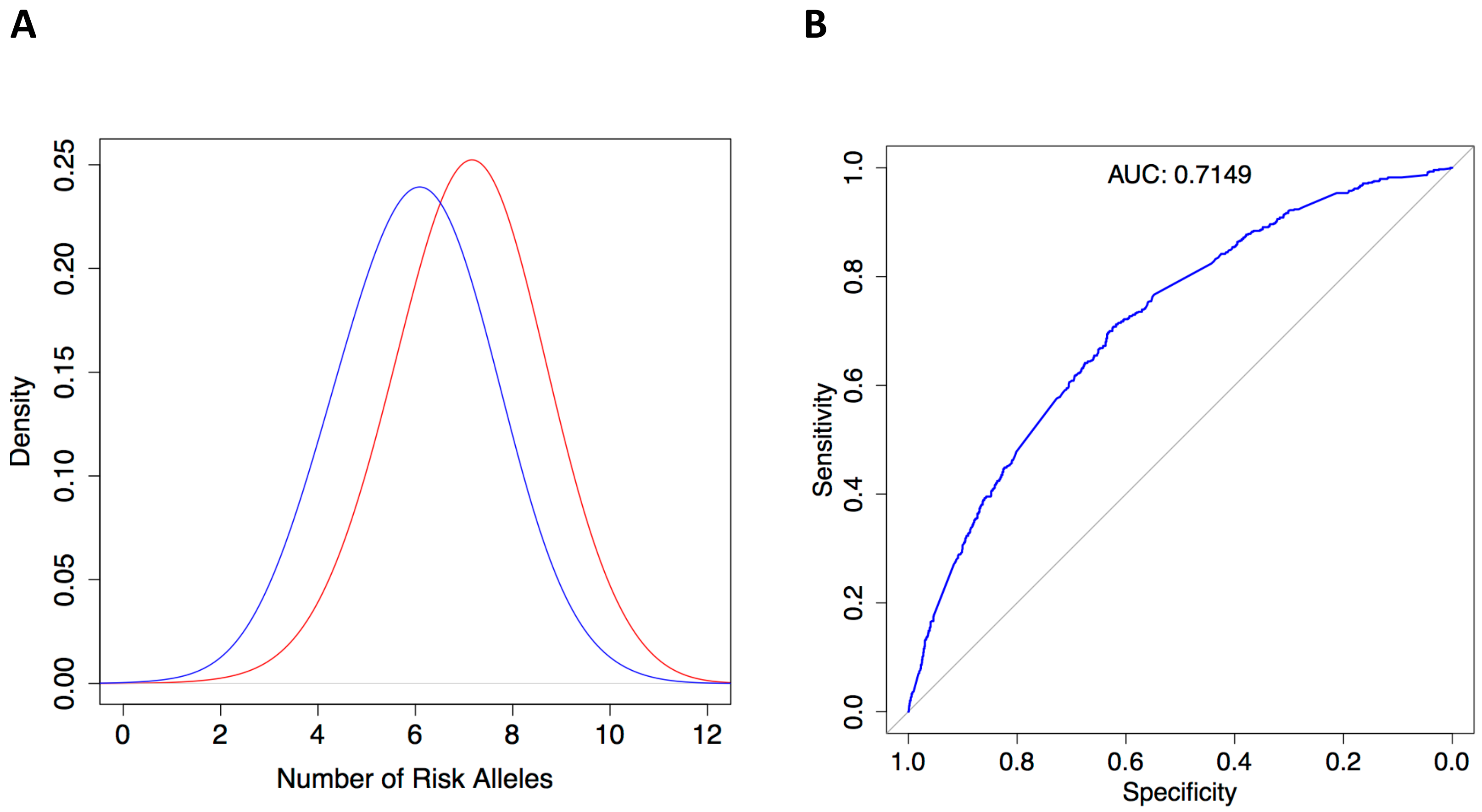

Supplementary Figure 13. Plots of the first 2 principal components showing the matching of cases (red) and controls (blue) across the 3 analytical sets: (A) published set, (B) NCI/Curie set and (C) CCSS set.

A

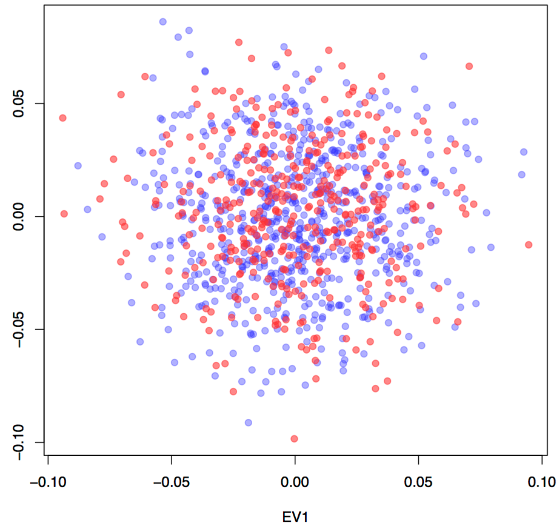

B

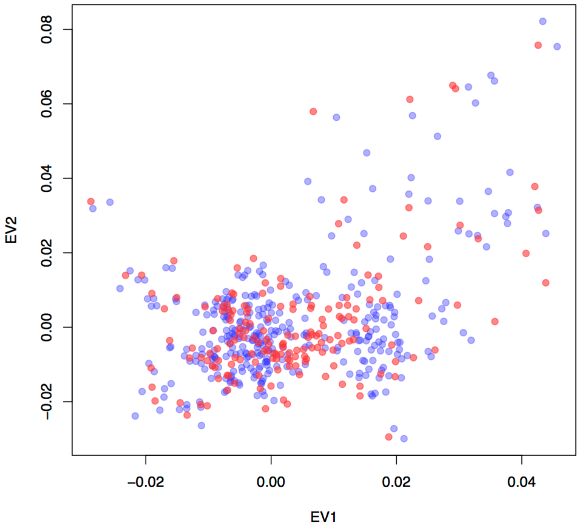

C

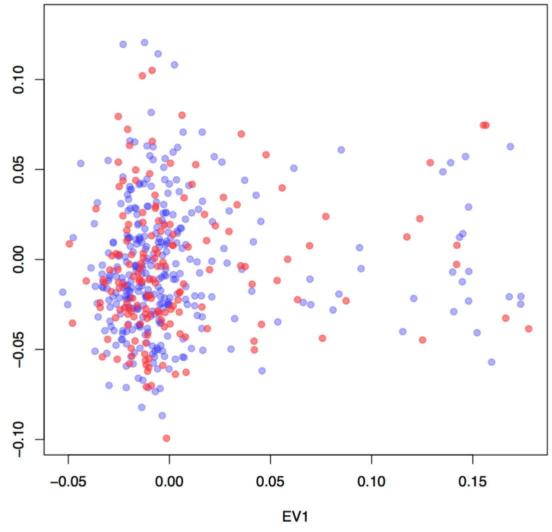

Supplement: Supplementary file 1 — Supplementary Information [file 41467_2018_5537_MOESM1_ESM.pdf]
